# Covid-19 in hospitals: Studying influencing factors through agent-based modelling

**DOI:** 10.1371/journal.pone.0326350

**Published:** 2025-06-18

**Authors:** Philippos Michaelides, Ştefan Sarkadi

**Affiliations:** 1 Department of Microeconomics and Public Economics, School of Business and Economics, Maastricht University, Maastricht, The Netherlands; 2 Department of Informatics, King’s College London, London, United Kingdom; University of Health Sciences, Beyhekim Training and Research Hospital, TÜRKIYE

## Abstract

Hospitals are highly dynamic environments where Covid-19 is highly transmissible if effective measures are not taken. General preventive policies are not necessarily effective; however, agent-based modelling can offer a way to tailor policies in such specific settings. In this paper, we develop an agent-based model to simulate a hospital as a multi-agent system and examine the influence of well-established Covid-19 factors on transmission, based on the Susceptible-Exposed-Infected-Recovered epidemic model. These factors include the mask efficacy, the mask-wearing percentage, the vaccination percentage, the room ventilation, the distancing rule, and the screening policy. This study, conducted in 2023, simulates agent interactions in a hospital setting using Python. We apply different parametrisations and employ a local sensitivity analysis to isolate the effect of each factor on transmission. We perform statistical analysis using independent two-sample t-tests, with a significance threshold of *p*<0.05. When worn by the entire population, N95 masks effectively mitigate transmission. They are more effective than surgical masks and substantially more effective than not wearing masks. Surgical masks, especially when worn by all individuals, also have a significant impact. Vaccination is highly effective, particularly when the entire population is vaccinated, and becomes critical when mask protection is weaker. Ventilation and distancing show minimal impact when N95 masks are used entirely within the hospital; alternatively, their effect is noteworthy but not highly decisive. Finally, the screening policy substantially affects transmission when a high percentage of entrants are tested, especially when surgical masks or no masks are worn. The findings highlight the need for prioritising mask-wearing and vaccination compliance, combined with a comprehensive screening policy. The model is highly parameterisable and can be adjusted to simulate other hospital settings or other infectious diseases, serving as a decision-making tool. Future studies could explore the combined effects of multiple interventions and validate the model with empirical data.

## Introduction

Counting five years since the first appearance of the Covid-19 pandemic (henceforth ‘Covid’ or ‘Virus’ or ‘Pandemic’), an unprecedented crisis to which humanity found it challenging to adjust, our lives have been gradually returning to their pre-pandemic state. However, the delayed reaction of governments and the World Health Organization (WHO) in instantly and directly combating the Virus, has resulted in the death of approximately 7 million people out of roughly 800 million positive cases worldwide.

The initial response to the Pandemic entailed implementing a range of non-pharmaceutical interventions (NPI) based on previous pandemic experiences and studies. These included public health measures such as social distancing, lockdowns, and mask-wearing. However, early medical research initially focused on the Virus characteristics, such as transmission means, contagiousness, incubation period and variances. As a result, more data became available, and the measures were updated accordingly, primarily based on their effectiveness. The evolving understanding of the factors influencing transmission, and mainly the vaccine distribution, resulted in more effective measures being implemented, and the Pandemic is now under control.

Yet, research into Covid continues to this day in an attempt to understand better its characteristics and factors influencing its spread. This is essential for ensuring that the most effective measures will be implemented in the case of future outbreaks, while guaranteeing that only necessary and truly protective measures are taken, and people are not subjected to constraints and limitations that do not ultimately protect them. Several fundamental medical research papers serve as the basis for subsequent mathematical and agent-based modelling studies. These latest studies aim to simulate the Covid spread within specific environments and situations to evaluate or even propose new customised suggestions for prevention.

Previous agent-based modelling research has been critical in understanding the efficacy of various control strategies in schools. Baccega *et al*., for example, used a model to evaluate active preventive strategies in a typical school environment during the Pandemic. Their research aimed to identify the most effective control strategy for mitigating the spread and avoiding extensive school closures while adjusting to disease spread [1]. In the same spirit, A. Fouad *et al*. simulated the routine of students and teachers in a typical elementary school, evaluating the effectiveness of preventive measures through the application of different school management policies [2]. Macalinao *et al*. conducted another study in which they simulated a typical classroom in the Philippines to investigate the effects of human interactions on Virus transmission in schools [3]. Furthermore, Phillips *et al*. simulated and studied Covid transmission in childcare centres, primary schools and households. In order to conduct an evidence-based review of plans to reopen childcare centres in Ontario, Canada, during the Pandemic, they investigated and analysed various factors that could influence and potential strategies that could mitigate the transmission [4].

In addition to schools, many other dynamic internal environments have been studied. For instance, Ying *et al*. have developed a model for evaluating the effectiveness of mitigation strategies in supermarkets. Their model considers customer movement and a transmission model determining if a given time spent close to infected individuals could transmit the Virus. Using this model, the impact of various mitigation strategies on reducing human-to-human transmission in the highly dynamic environment of a supermarket was effectively quantified [5]. More recently, Qiao *et al*. assessed the transmission risk and the efficacy of face coverings, vaccination, ventilation, social distancing, and isolation in the dynamic and labour-intensive environment of a construction site [6]. Finally, Ciunkiewicz *et al*. suggested a configurable model to simulate Covid spread in small, highly localised, and variable environments, namely offices, campuses, or long-term care facilities. The main objective was to provide actionable insights by forecasting transmission while considering epidemiological parameters, airborne viral spread, vaccination, and mask-wearing [7].

At the same time, researchers have used agent-based models to evaluate the effectiveness of preventive measures implemented by governments and local councils in cities and countries. For example, Hoertel *et al*. evaluated the effect of post-lockdown interventions, such as social distancing and mask usage, based on the cumulative disease incidence, mortality, and ICU-bed occupancy in France [8]. Similarly, Gharakhanlou *et al*. studied the effectiveness of measures such as school and educational centre closures and social distancing against the Covid spread in Urmia city, Iran [9]. Additionally, Shamil *et al*. simulated the spread of Covid in Fork County and New York City, USA, to evaluate the effectiveness of different governmental measures, such as early lockdown and contact tracing via smartphones [10]. Aleta *et al*. assessed the effectiveness of measures such as social distancing, level of testing, contact tracing, and household quarantine in the Boston area of the USA [11]. Finally, Wilder *et al*. applied agent-based modelling to study the impact of demographic structure on Covid transmission in Hubei, Lombardy, and New York City, while also assessing the effectiveness of preventive measures [12,13].

Despite the widespread use of agent-based modelling in both indoor and outdoor environments, our understanding of how Covid spreads—and how specific, controllable factors influence transmission within hospitals—remains limited. Hospitals are particularly dynamic environments due to routine patient admissions, discharges, and visits, resulting in a continuously changing and diverse population. They also house individuals at significantly higher risk, such as elderly or severely ill patients, leading to increased mortality in the event of an outbreak. These conditions require different—typically richer and more complex—simulation approaches than those used for schools, offices, or public spaces. Broader Covid challenges—such as caregiver burden [14], pre-hospital emergency issues [15], and hospital management difficulties [16]—also underscore the complexity of healthcare settings and the value of tailored approaches.

This study addresses this critical gap by developing an agent-based model (ABM), based on Baccega [1] and specifically tailored to hospital settings, to simulate Covid transmission, assess the impact of key influencing factors, and identify effective management and policy interventions.

Our paper makes several contributions. While most of the influencing factors have already been studied in the context of medical research and mathematical analyses, the value of this work lies in applying and adapting that knowledge to the hospital setting. Covid transmission has been studied for isolated single hospital units and simple interactions [17], and our ABM builds upon this research by expanding to realistically encompass a complete, highly dynamic, and diverse hospital environment. It accounts for many of its population and interaction dynamics by simulating and thus considering complex human behaviours and particularities. It is also parameterisable and well-documented, allowing for adjustments (with minor code changes) to cover other diseases or to be distributed to various hospitals worldwide, tailored to their specific settings and needs. That is, it could serve as a valuable, practical, and efficiently running multi-agent decision-making tool for governments or hospital management systems. Such a tool would benefit citizens around the world by enabling their governing institutions to reach conclusions tailored to specific environments and resource availability in the health and public safety domain. This would lead to more effective public health strategies and enhance the quality of healthcare delivery and social well-being for diverse populations.

The two main research questions that we seek to answer in this paper are: RQ1: *How and to what extent do well-known factors influence transmission in an indoor hospital environment?* and RQ2: *What management policies and individual practices might be able to mitigate the spread of Covid in a hospital and reduce the confirmed positive cases rate?*

We aim to answer RQ1 by modelling and simulating different parametrisations (henceforth ‘scenarios’) with various combinations of factor values using local sensitivity analysis. We are interested in the type and efficacy of masks, the percentage of mask wearers, the percentage of people vaccinated, the hospital room ventilation, the distancing rule enforced, and the screening policy (i.e., the percentage of incoming individuals being tested). For each factor, we aim to investigate its correlation (positive or negative) with the Virus spread and its magnitude of impact.

Hospital management can influence these factors directly, such as by determining the desired room ventilation, or indirectly, by encouraging or enforcing behaviours such as mask-wearing through the relevant measures. Therefore, by answering RQ1, we identify the most effective management policies and individual practices to limit the spread of Covid, providing conclusions to answer RQ2.

Overall, all considered influencing factors prove important in the critical hospital setting, but mask-wearing, vaccination, and screening are the most decisive. In any case, the influence of each factor is quantified, allowing for the recommendation of specific mitigation policies.

## Methods

### Code availability

The source code of the model can be found in the supplementary material. See S1 Appendix for the source code structure and model usage guidelines.

### Hospital setting

We model a typical small to medium-sized hospital, emphasising the key facilities, stakeholders, operations, and policies.

**Fig 1:**
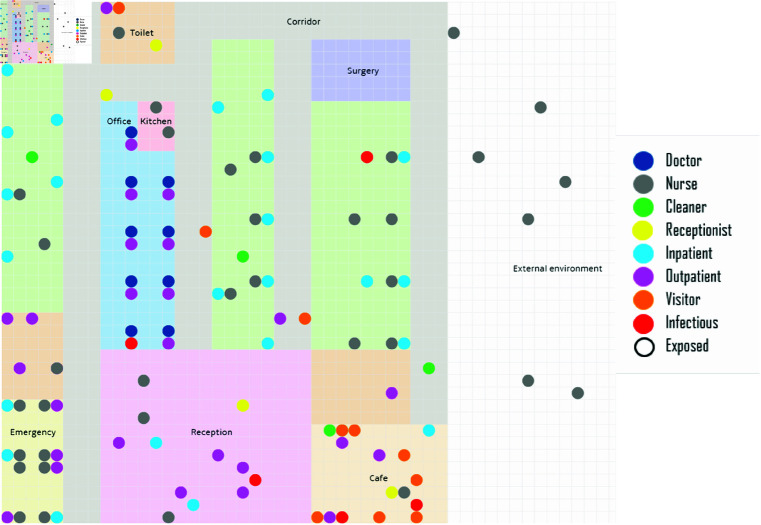
Hospital floor plan as assumed in the model. Each grid cell is assumed to be 1 *m* in width and height. Therefore, the building measures 36 *m* in length, 42 *m* in width, has an assumed height of 3 *m* and consists of several rooms. The rooms are visualised as rectangles (as their labels indicate) and humans as dots, of different colours.

As for the layout and structure of the hospital building, apart from the doctor offices and patient rooms with private toilets (double or single, totalling 32 beds), the building includes emergency rooms, surgery rooms, and toilet facilities. Additionally, the hospital features a spacious reception area, a cafe, a small staff kitchen, and the necessary corridors. Regarding the individuals (stakeholders) who utilise the hospital, we can identify three distinct groups (roles): a) patients, who can be either inpatients or outpatients; b) staff, including doctors, nurses, cleaners, and receptionists; and c) visitors. Each group is modelled by considering its diverse characteristics, ensuring that all their dynamic interactions are realistically simulated. The population within the hospital is dynamic, with permanent changes observed in the patient and visitor population due to scheduled medical visits (doctor appointments), emergency medical visits, visits from visitors, admissions and discharges. However, the staff population remains constant, with only temporary changes due to staff shifts. Hospital operating hours are between 07:00 and 19:00 and include admissions, discharges, doctor appointments, emergency medical visits, surgical procedures, visiting hours, and cafe availability, with most hospital staff on duty. Outside these hours, only emergency patient admissions and medical visits are permitted (at lower rates), while the hospital operates with a reduced staff. Finally, we take into account the possibility that an individual entering the hospital could be infected, based on a fixed probability assumed to be 2%, expecting that in all these cases, they are in the Covid stage able to transmit the Virus (they are infectious), to account for the worst-case scenario. Fig 1 visualises how the hospital setting and its agents are simulated. The design decisions represent a simplified version of established practices that are followed in real-life cases, and they are fully parameterisable so that the model can adjust and capture various situations. For additional details on the design and assumptions concerning the environment, population, policies, and human behaviour, as well as the external environment, see S2 Appendix.

### Covid transmission

#### Investigated factors.

As mentioned before, our aim is to investigate and quantify the influence of five widely recognised factors on Covid spread.

**Mask-wearing:** One of the primary factors we are studying is mask-wearing. Many research studies have investigated the importance of wearing a mask and concluded a negative correlation with Virus transmission, proving that masks significantly reduce the risk of infection [18]. We are interested in quantifying this negative correlation, considering two key factors: the efficacy of the mask type used and the mask-wearers percentage. We assume the masks are worn correctly, with any improper fit considered as not wearing a mask at all. Furthermore, we assume adherence to all health protocols, including the recommended change frequency and wearing procedure. These are necessary to ensure that the designed efficacy equals the actual one that practically affects transmission in real-life situations. We also expect a uniform mask type to be consistently used by all mask-wearing individuals within each simulation, in all rooms and regardless of their role. Finally, we assume that the efficacy of exhalation (filtering exhaled particles, reducing transmission risk to others) is equal to the efficacy of inhalation (filtering inhaled particles, reducing the wearer’s risk). We only account for the N95 medical respirator (henceforth ‘N95’) and medical grade procedure (henceforth ‘surgical’) mask types, with efficacy levels of 99% and 59%, respectively [19].

**Vaccination:** Previous research also demonstrates that vaccines significantly reduce the infection risk and mitigate transmission [20]. In light of this, our study focuses on quantifying the negative correlation between vaccination and Covid spread by considering the percentage of vaccinated individuals. Given that the two most widely used and accepted vaccines (Moderna and Pfizer) have similar efficacy levels, we do not differentiate between vaccine types. Instead, we assume a single vaccine type with a given efficacy level. We assume that individuals are either fully vaccinated, with an efficacy level of 95% [21] or not vaccinated at all, with an efficacy level of 0%. We assume the full vaccination efficacy level without considering potential influencing factors.

**Ventilation:** Another factor under consideration is ventilation in common hospital areas and rooms. Several studies again suggest that ventilation reduces the viral load in an indoor environment [22], and the aim is to quantify the magnitude of the impact in the hospital setting. This is under the assumption that the hospital being modelled exclusively relies on air exchange (ventilation) rather than filtering and cleaning the indoor air (air purification or sterilisation). We also assume mechanical and not natural ventilation, using Heating, Ventilation, and Air Conditioning (HVAC) systems. This is important to control the air changes performed and evaluate the influence of different system configurations and ventilation policies on the transmission. More specifically, we measure how many times per hour the air in a given space is replaced with fresh air (Air Changes per Hour—*ACH*) [23], assuming that the mechanical systems are functioning properly as designed. We finally assume the same air ventilation rate in all rooms every time.

**Distancing:** Distancing is considered another significant factor in mitigating Covid transmission [24]; one of the primary governmental measures during the recent outbreaks was enforcing a minimum social distance indoors and outdoors. Our model simulates different distancing rules (minimum allowed distance between individuals) to investigate the magnitude of their impacts, disregarding the possibility of people violating the rule. However, as expected, this rule is not followed in unavoidable situations, such as between staff and patients in patient, emergency, and surgery rooms. Based on the model representation, individuals in the same grid cell are assumed to be distanced by 0.5 *m*. When located in two adjacent cells, including diagonals, their distance is 1 *m*. With one empty grid cell between them, the distance is 2 *m*; with two empty grid cells, the distance is 3 *m*, and so on.

**Screening policy:** Finally, we investigate how and to what extent different screening policies affect Virus transmission in the hospital, considering the great emphasis given in this specific NPI. The various simulated policies differ regarding the percentage of incoming individuals (patients, staff members and visitors) tested, assuming only one testing method with a false negative ratio (proportion of negative test results that are incorrect and should have been positive) of 5% [25]. We do not, however, account for the established policy of hospitals to regularly test admitted inpatients or staff members for quarantine purposes. Including and investigating this factor would overload the experimental design and increase the complexity of the model, requiring special handling of positive cases.

To study the influence of the screening percentage, assuming that individuals who test positive are not allowed to enter, we adjust the probability of entering the hospital while being infectious as follows:


$$P=P(1-t_{p}t_{a}),$$

where *P* is the probability (2%), *t*_*p*_ is the percentage of entrants we test (varies in each scenario), and *t*_*a*_ is the test accuracy (95%).

Several studies have suggested that temperature and humidity can inversely affect the spread of Covid by influencing the production of droplet nuclei [26–28]. However, other studies have found no significant positive or negative correlation [29,30]. Due to the mixed and sometimes conflicting findings, and given that their impact is still not fully understood, we do not investigate these factors. In any case, hospital temperature and humidity levels are typically maintained at a standard level, so management has limited control over them.

#### Epidemic model.

**Fig 2:**
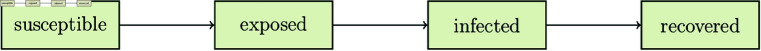
SEIR model. *Susceptible* individuals first become *exposed* when they get infected, and then *infected* when infectious. Eventually, they recover and become *recovered* and immune.

We use the well-known Susceptible-Exposed-Infected-Recovered (SEIR) model [31] (illustrated in Fig 2). *Susceptible* individuals are those able (vulnerable) to contract the Virus, *exposed* have been infected but are not yet infectious, *infected* have been infected and are infectious, and *recovered* have become immune and are neither infected nor infectious. We study the impact of the investigated factors based on the *exposed* and *infected* populations.

Every *susceptible* individual can contract the Virus after contact with an *infected* one. This is given by a certain probability based on the type of contact. In an infection case, the *susceptible* individual becomes *exposed* immediately, right after the contact. *Exposed* individuals become *infected* when they become infectious. To define the infection to infectiousness period (henceforth ‘latent period’), we use the mean value of 5.5 days as estimated in a recent study [32]. Therefore, the *exposed* individual becomes *infected* and is capable of transmitting the Virus 5.5 days after exposure, without considering potential influencing factors. We expect an *infected* individual to continue to transmit the Virus throughout the entire period until they have transitioned to the *recovered* state. We assume asymptomatic *exposed* and *infected* individuals to model the worst-case scenario, where no one is quarantined. An *infected* individual eventually (except in the event of a death) becomes *recovered*. Given that the WHO initially recommended quarantine at home for 14 days from the last exposure [33], and despite most people being no longer infectious after day 10 [34], we define the infected and infectious period (henceforth ‘quarantine period’) as 14 days from the exposure time. That is, 14 days after the day of the individual being *exposed*, they reach the *recovered* stage. Finally, a *recovered* individual can become *susceptible* again after a certain period (henceforth ‘waning immunity period’) [35]. This is because of the gradual decrease in the protective antibodies developed after an infection. To define this period, we adopt the results of a recent study, based on which the interval between reinfections is approximately a year [36]. Considering the relatively shorter duration of the simulation, *recovered* individuals remain immune until the end of the simulation.

#### Transmission drivers.

We distinguish between close-range and airborne transmission and account for these two drivers of exposure: **a.** respiratory airborne aerosol transmission (vulnerable in the same room with infectious), and **b.** direct person-to-person (close) contact between vulnerable and infectious transmission. For more details on how Covid is transmitted, see S3 Appendix.

**Respiratory airborne aerosol transmission:** Infectious particles (respiratory droplets and aerosols) contain virions that, if inhaled, can transmit Covid. The probability (individual risk) of infection depends on the number of virions needed on average to infect a vulnerable individual and the total number of virions inhaled. It is assumed that virions inhaled by individuals are reset to zero upon exiting the hospital, considering that human behaviour and Covid-related parameters are uncontrolled outside the hospital environment. The probability is given by the following equation using the dose-response model [37,38]:


$$P=1-e^{-[\frac{(1-v_{eff}\;v_{fr})N}{N_{0}}]},$$

where $v_{eff}$ is the vaccine efficacy, $v_{fr}$ is the percentage of vaccinated people, *N* is the total number of virions inhaled over a time period *T*, and *N*_0_ is the number of virions needed on average to infect an individual. This probability determines whether an individual gets infected and is only triggered when they leave a room, given that it represents the cumulative risk of staying in a specific room for a given period and with a calculated concentration of virions.

While vaccine parameters $v_{eff}$ and $v_{fr}$ vary at different simulated scenarios, *N*_0_ is a constant model parameter. We adopt the value proposed in Watanabe *et al*. [37], and thus $N_{0}=4.1\;\times \;10^{2}$. The total number of virions inhaled until the time instance *t*, is given by the following equation [39,40]:


$$N(t)=N(t_{0})+(1-m_{ieff}m_{fr})I_{r}\int_{t_{0}}^{t}C(\xi )\;d\xi ,$$

where *N*(*t*_0_) is the accumulated amount of virions inhaled until *t*_0_ (*t*>*t*_0_), *m*_*ieff*_ is the inhalation mask efficacy, *m*_*fr*_ is the percentage of people wearing the mask correctly, *I*_*r*_ is the inhalation rate (volume of air inhaled over a given time period [41]), and *C*(*t*) is the local virions concentration (of the room where the individual is located and breathing) at time instance *t*.

Mask parameters *m*_*ieff*_ and *m*_*fr*_ again vary depending on the scenario, while inhalation rate *I*_*r*_ primarily depends on the activity and posture (e.g., standing, sitting, quiet breathing, talking, shouting, heavier activities like running, climbing and so on) [38]. For this study in the hospital setting, we assume normal activity and thus an average inhalation rate between 0.521 and 2.5 *l*/*s* (we choose randomly to cover both ‘sitting/speaking/breathing’ and ‘standing/exercise’ activities respectively) [39,42]. We ignore unusual activities (e.g., running during an emergency) and other possible factors like sex, age, fitness level, and medical conditions. The local virions concentration of a room at time instance *t* is given by the following equation [39,40]:


$$C(t)=\frac{n_{inf}(1-m_{eeff}m_{fr})G_{r}}{VR}+[C(t_{0})-\frac{n_{inf}(1-m_{eeff}m_{fr})G_{r}}{VR}]e^{-R(t-t_{0})},$$

where *n*_*inf*_ is the number of infectious individuals in the room of volume *V*, *m*_*eeff*_ is the exhalation mask efficacy, *G*_*r*_ is the aerosols generation rate (aerosols emitted per time unit), and *C*(*t*_0_) is the local virions concentration at time instance *t*_0_ (*t*>*t*_0_). R=λ+κ+υ tries to simulate the removal of virions from the air due to the viral decay rate λ (rate of decline of free viruses [43]), gravity settling (of aerosols) rate κ, and ventilation rate υ [39,40], assuming no sterilisation.

Room parameters *V* and *n*_*inf*_ take values based on each state of the simulation, and mask parameters *m*_*eeff*_ and *m*_*fr*_ vary among the different scenarios. Gkantonas *et al*. concluded that aerosols generation rate *G*_*r*_ depends on the aerosol particle’s upward air velocity, cut-off diameter, and viral load. They extensively discuss and explain these and other possible influencing factors, and estimate different generation rates for various combinations [39]. For our hospital model, we assume a zero air velocity, a cut-off diameter of 20 μm and a viral load of $10^{9}\;copies/ml$, as also Baccega *et al*. adopted in their school model [1]. Therefore, and based on Gkantonas’ estimations, we assume $G_{r}=0.589\;PFU/s$. Finally, to address the removal of virions from the air, we assume a viral decay rate of $\lambda =0.636\;h^{-1}$ [39,44] and a gravity settling rate of $\kappa =0.39\;h^{-1}$ [40]. Ventilation rate υ depends on the scenario simulated.

**Direct person-to-person (close) contact transmission:** In a case of close and direct contact between a vulnerable and infectious individual, the probability of respiratory airborne aerosol transmission, as calculated above, still holds and defines whether or not the vulnerable individual will become infected. However, because of the closer contact, another independent probability exists (triggered per minute) to capture the increased danger. This probability is given by the following formula [1,8]:


$$P=C_{r}\frac{D}{A},$$

where *C*_*r*_ is the contamination risk, *D* is the duration of contact (in minutes) and *A* is the area of contact.

The referenced papers suggest a contamination risk of 0.024, reduced by roughly 50% when a mask is worn, without considering the mask efficacy (type) or vaccination status. For our study, we assume the contamination risk to be calculated as follows:


$$C_{r}=0.024*(1-m_{ieff}m_{fr})*(1-v_{eff}v_{fr}),$$

so that mask efficacy, mask-wearing probability, vaccination efficacy, and vaccination probability are considered.

The duration is always 1 *min* since this probability is calculated and triggered every simulated minute (throughout the total duration of exposure), while the area is suggested as a fixed number of $4.41\;{\text{m}}^{2}$ around the infectious individual. We, therefore, consider close contact as anyone within 1 *m* of an infectious individual, using an area of $4\;{\text{m}}^{2}$ for simplicity. In such a case, vulnerable individuals face an additional risk based on this probability, calculated and applied independently for each infectious individual within this radius, based on the duration of exposure with each one, and irrespective of their exact distance.

### Model verification

Given the complex behaviour our model tries to simulate and the complicated and long code developed (see S4 Appendix for all model implementation details), verification and testing techniques are put in place to ensure correctness.

The non-deterministic nature of our system challenges its verification, as no expected outcomes can be defined to compare with the actual ones. While current approaches are being developed for verifying and validating stochastic ABMs [45], we are limited to uncovering blatant abnormalities for our model. Namely, we are limited to exceptions raised in the context of tests performed during the simulation, so implementation faults that lead to blatantly unexpected behaviour and contradict our requirements show themselves (e.g., negative probability or population). We also run our model with boundary values to test some corner cases, such as ensuring no infections occur when zero of the hospital’s initial population or entrants are infectious.

Here, we focus on the adjustment to verify our implementation of the respiratory airborne aerosol transmission equations against those proposed by Gkantonas *et al*. [39]. We run a deterministic scenario where a hospital room is isolated, and all relevant parameters are controlled. This allows us to compare our model results with those of the referenced model under the same conditions, ensuring the implementation’s correctness.

**Fig 3:**
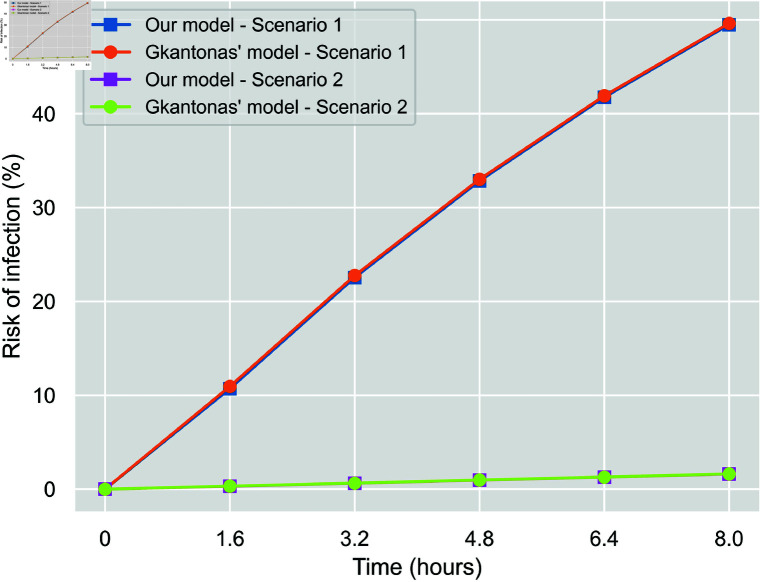
Model verification. Comparison of cumulative individual infection risk per time spent in a room between our model and Gkantonas’ model. *Scenario 1*: No mask, ventilation rate of 2 *ACH*, room volume of $36\;{\text{m}}^{3}$, and *Scenario 2*: 100% mask-wearers, ventilation rate of 10 *ACH*, room volume of $72\;{\text{m}}^{3}$.

Fig 3 compares the two models’ results concerning the cumulative individual infection risk (probability) per time spent inside a room of a given volume. We keep a fixed number of two individuals (one infectious and one vulnerable), a fixed inhalation rate of 0.521 *l*/*s*, and set the values for aerosols generation rate, decay rate and gravity settling rate at 0.589 *PFU*/*s*, $0.636\;{\text{h}}^{-1}$ and $0.39\;{\text{h}}^{-1}$ respectively, as adopted for our model in general. The number of virions needed to get infected remains at $4.1\;\times \;10^{2}$, and the viral load at $10^{9}\;copies/ml$. Since Gkantonas’ model does not include the effect of vaccination, we ignore this factor entirely, assume a fixed mask efficacy of 59%, and indicatively compare for two scenarios varying mask-wearing, ventilation rate and room volume. We can observe identical infection probabilities, concluding a correct implementation of the transmission probabilistic approach.

### Simulation

Whether an agent becomes infected or not (agent condition), as well as the agent’s decisions and behaviours, including initial location, movement within and between rooms, duration of stay in rooms, admissions/discharges, medical visits, visitor visits, mask-wearing, vaccination status, and more, are all random and probabilistic. In all cases, all possible outcomes are mutually exclusive and exhaustive, resulting in a discrete probability distribution [46]. This introduces uncertainty and variability into the simulation process, and to account for this, multiple simulation runs are performed for each scenario. Employing a total of 30 simulation runs was sufficient to stabilise the results of each scenario, as shown by gradually increasing the number and observing their convergence. Given the Covid timeframes (latent, quarantine and waning immunity periods), we define a simulation to last for one month, starting on 1 July at 07.00 and ending on 1 August at 07.00. The initial state of the model, before any actions or movements are executed, can be described as follows: we define 25 inpatients, 30 outpatients, and 10 visitors to be initially located inside the hospital. Also, all staff members of the first shift are assumed to perform their duties normally. For more information on the simulation, see S5 Appendix.

### Metrics and statistics

The metrics obtained in each simulated run are the following:

1. Cumulative cases: a running count of all Covid cases reported in the hospital over the one-month simulated time, including infectious entrants.
2. Cumulative generated cases: cumulative cases generated inside the hospital (infectious entrants are excluded). We are also interested in the cumulative generated cases per day.
3. Cumulative exogenous cases: cumulative cases considering only infectious entrants.
4. Daily generated cases: a count of daily (07.00 - 07.00) generated cases (infectious entrants are excluded).

To compare the various scenarios and reach some conclusions about the investigated factors and measures, these metrics are analysed and compared considering the cumulative population of the hospital during the simulation period. This is very important, given that relying solely on the absolute number of cases might be misleading.

Given the metrics retrieved, for each simulation run, we calculate the following statistics:

1. Average daily generated cases: the average number of cases generated inside the hospital per day.
2. Transmission ratio: the number of cases generated inside the hospital, resulting from each infectious entrant. Calculated as the cumulative generated cases divided by the cumulative exogenous cases.
3. Cumulative cases percentage: percentage of cumulative cases in relation to the cumulative hospital population.

### Experimental set-up

Table 1 illustrates the 43 scenarios simulated. Our set-up is organised in two parts: a. investigating the effect of mask type and mask-wearing percentage and b. investigating the effect of the rest factors, considering the mask type. A local (one at a time) sensitivity analysis is employed; only one parameter varies in each scenario while keeping the rest fixed. For the fixed parameters, as shown in the table, the respective average values are used.

**Table 1:** Experimental set-up.

	Mask type			Mask-wearing (%)				Vaccination (%)			Ventilation (*ACH*)			Distancing (*m*)			Screening (%)		
	N95	Surgical	No	0	50	75	100	0	50	100	4	8	12	0	1	2	0	50	100
**1**			x	x					x			x			x			x	
**2**	x				x				x			x			x			x	
**3**	x					x			x			x			x			x	
**4**	x						x		x			x			x			x	
**5**		x			x				x			x			x			x	
**6**		x				x			x			x			x			x	
**7**		x					x		x			x			x			x	
**8**	x						x	x				x			x			x	
**9**	x						x		x			x			x			x	
**10**	x						x			x		x			x			x	
**11**	x						x		x		x				x			x	
**12**	x						x		x			x			x			x	
**13**	x						x		x				x		x			x	
**14**	x						x		x			x		x				x	
**15**	x						x		x			x			x			x	
**16**	x						x		x			x				x		x	
**17**	x						x		x			x			x		x		
**18**	x						x		x			x			x			x	
**19**	x						x		x			x			x				x
**20**		x					x	x				x			x			x	
**21**		x					x		x			x			x			x	
**22**		x					x			x		x			x			x	
**23**		x					x		x		x				x			x	
**24**		x					x		x			x			x			x	
**25**		x					x		x				x		x			x	
**26**		x					x		x			x		x				x	
**27**		x					x		x			x			x			x	
**28**		x					x		x			x				x		x	
**29**		x					x		x			x			x		x		
**30**		x					x		x			x			x			x	
**31**		x					x		x			x			x				x
**32**			x	x				x				x			x			x	
**33**			x	x					x			x			x			x	
**34**			x	x						x		x			x			x	
**35**			x	x					x		x				x			x	
**36**			x	x					x			x			x			x	
**37**			x	x					x				x		x			x	
**38**			x	x					x			x		x				x	
**39**			x	x					x			x			x			x	
**40**			x	x					x			x				x		x	
**41**			x	x					x			x			x		x		
**42**			x	x					x			x			x			x	
**43**			x	x					x			x			x				x

Scenario 1 assumes no mask and fixed average values for the other factors. Each Scenario 2-7 simulates a different mask-wearing condition regarding mask type and wearing percentage while maintaining a fixed average value for other factors. Scenarios 8-10, 20-22, and 32-34 vary vaccination percentage assuming N95, surgical and no mask, respectively, while maintaining a fixed average value for other factors. Similarly, Scenarios 11-13, 23-25, and 35-37 refer to ventilation rate, Scenarios 14-16, 26-28 and 38-40 relate to the distancing rule, and Scenarios 17-19, 29-31 and 41-43 to the screening percentage. For Scenarios 8-43, when the mask is used, a 100% wearing percentage is assumed.

**a. Mask type and wearing percentage:** We only account for the N95 and surgical mask types, with efficacy levels of 99% and 59%, respectively. We also consider wearing percentages of 0%, 50%, 75% and 100%.

*Experiment I:* To study the mask impact, we use a baseline scenario in which no mask is worn, which is then independently compared with scenarios where the mask is used under different conditions (in terms of mask type and wearing percentage) to quantify mask impact under each of these conditions.

*Experiment II:* In addition, the two mask types are directly compared to each other under all three conditions of mask-wearing percentage. This is important to comprehend whether the higher efficacy of the N95 mask significantly reduces Covid transmission, compared to the surgical mask, and to quantify this difference in effect in relation to the essential factor of wearing percentage.

*Experiment III:* Finally, for each mask type individually, we directly compare the impact of wearing percentages to observe the effects of higher percentages while using a specific mask type.

**b. Remaining factors:** To study the effect of the remaining factors (i.e., vaccination, ventilation, distancing, and screening policy), we examine under which factor-related conditions (i.e., vaccination percentage changes, ventilation rate changes, distancing rule changes and screening percentage changes) changes in Covid transmission are observed. We do this by comparing scenarios within each factor, for both N95 and surgical masks (maintaining a fixed wearing percentage of 100% to study the impact in an absolute case) and for no mask worn as well.

For all the experiments, we conduct statistical hypothesis tests (independent two-sample t-tests), aiming to determine whether there is a statistically significant difference between the statistics (means) resulting from the different scenarios. We make our decision based on the average daily generated cases of each scenario. For more details on the simulated scenarios, experiments and hypothesis tests conducted, see S6 Appendix.

### Model parameters

The Covid parameters and the values used for the investigated factors are summarised in Table 2.

**Table 2:** Covid parameters representing real-life characteristics as found in the literature.

Parameter	Value
Probability of entering as infectious	2 %
Surgical mask efficacy	59 %
N95 mask efficacy	99 %
Vaccine efficacy	95 %
Inhalation rate	0.521 - 2.5 *l*/*s*
Virions needed on average to infect	410
Aerosols generation rate	0.589 *PFU*/*s*
Decay rate	$0.636\;{\text{h}}^{-1}$
Gravity settling rate	$0.39\;{\text{h}}^{-1}$
Covid test accuracy	95 %
Latent period	5.5 days
Quarantine period	14 days
Waning immunity period	365 days
Mask type	N95 / surgical
Mask-wearing percentage	0 / 50 / 75 / 100 %
Vaccinated individuals percentage	0 / 50 / 100 %
Ventilation rate	4 / 8 / 12 *ACH* [47]
Distancing rule	0 / 1 / 2 *m*
Screening policy	0 / 50 / 100 %

The last six lines pertain to the investigated factors. All values are converted to the International System of Units (SI) for the calculations.

## Results

This section presents the simulation results obtained following the experimental set-up and performing the experiments discussed before. We selectively present and visually highlight the important metrics and statistics based on the means (and the standard deviations) of the total of 30 runs performed for each scenario. The full results, including intermediate results of each specific run and additional results not presented here, along with the details and results of all conducted hypothesis tests, can be found in the supplementary material.

### Mask-wearing

To investigate mask impact regarding type and wearing percentage, *Experiment I* was performed where all mask-wearing conditions (Scenarios 2-7) were compared to the baseline scenario of no mask (Scenario 1). All the t-tests returned very low p-values (lower than our alpha of 0.05), meaning there is a statistically significant difference in Covid spread between wearing and not wearing a mask for all mask types and wearing percentages. We therefore conclude that masks reduce Covid under all wearing conditions.

Based on the results summarised in Table 3, Covid spread is significantly reduced in all scenarios where the mask is worn, compared to the no-mask scenario, with this to be reflected in all related metrics and statistics. Namely, when the hospital population wears masks, regardless of the mask type and wearing percentage, the hospital experiences significantly fewer average daily and cumulative generated cases and, therefore, a smaller transmission ratio and cumulative cases percentage.

**Table 3:** Metrics and statistics for Scenarios 1-7.

	Scn.1	Scn.2	Scn.3	Scn.4	Scn.5	Scn.6	Scn.7
**Average daily generated cases**	24.7	11.9 (-52%)	5.1 (-79%)	0.1 (-100%)	16.6 (-33%)	13.0 (-47%)	9.2 (-63%)
**Cumulative generated cases**	819	392	168	3	553	427	302
**Cumulative cases^a^**	914	493	273	116	648	528	400
**Cumulative cases percentage**	10.9%	5.9%	3.2%	1.4%	7.7%	6.3%	4.8%
**Transmission ratio**	8.6	3.8	1.6	0.0	5.8	4.3	3.1

The numbers in parentheses express the percentage changes in average daily generated cases when switching from Scenario 1 to each of Scenarios 2-7. This table was previously published in our short related paper [48]. ^a^ Includes both generated and exogenous cases (infectious entrants).

As can be seen, not wearing a mask could infect more than 10% of the population, reporting around 25 daily generated cases. This is also reflected by the transmission ratio of about nine, meaning that for every single infectious entrant, nine vulnerable individuals are eventually infected. Mask-wearing is an effective measure, resulting in fewer generated cases and a lower transmission ratio, mitigating the spread inside the hospital even as infectious individuals continue to arrive at a constant rate. When worn at a low percentage (50%), the surgical mask shows a significant decrease in spread of around 30%. Higher percentages, especially with N95 masks, can reduce the spread by more than 50%. We highlight the impressive total absence of generated cases when 100% of the population wears N95 masks; the 113 infectious entrants lead to only three infections in 31 days (transmission ratio of just 0.03). All percentage changes are shown in Table 3.

**Fig 4:**
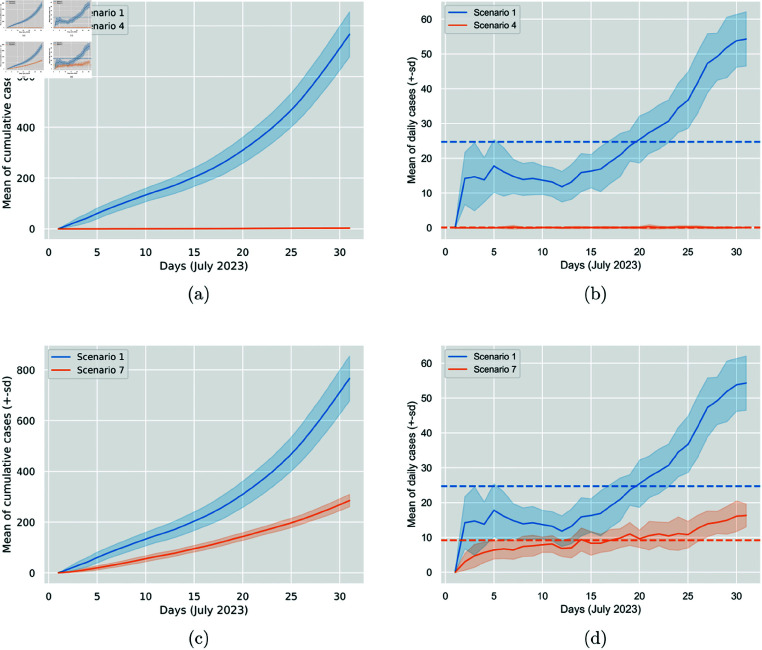
Comparison of the impact between N95 (Scenario 4) and surgical masks (Scenario 7) at 100% mask-wearing percentage. The figure displays the cumulative generated cases per day and the daily generated cases. In Sub-figures (a) and (c), the solid line represents the 30-run mean of the cumulative generated cases per day, and the colourful area around it is the standard deviation. In Sub-figures (b) and (d), the solid line represents the 30-run mean of the daily generated cases, the colourful area around it is the standard deviation, and the dashed line is the average. This figure was previously published in our short related paper [48].

Fig 4 visualises the comparative results between the baseline scenario and Scenarios 4 and 7, where the N95 and surgical masks, respectively, are worn 100%, and it blatantly shows the significant impact on the cumulative generated and daily generated cases.

For *Experiment II*, all Scenarios 2-4 were directly compared with all Scenarios 5-7, so the two masks are directly compared to each other under all three mask-wearing percentage conditions. All the t-tests performed for each comparison returned very low p-values, meaning that mask type significantly affects transmission for all mask-wearing percentages. While N95 masks are generally more effective than surgical masks, the wearing percentage plays an important role.

The metrics and statistics reported for each involved scenario are shown in Table 3. It can be observed that wearing N95 masks at a percentage of 75% or higher is more effective in mitigating the spread than wearing surgical masks, even if the surgical masks are worn by all participants. Also, the N95 mask type for 50% wearing percentage is significantly more effective than 50% surgical mask, and slightly more effective than 75% surgical mask. However, it has been proven more effective to use surgical masks at a wearing percentage of 100%, than using N95 at 50%. Table 4 summarises these conclusions while quantifying the effectiveness difference.

**Table 4:** Percentage changes in average daily generated cases when switching from surgical mask (Scenarios 5-7) to N95 mask (Scenarios 2-4).

	Scn.2	Scn.3	Scn.4
**Scn.5**	-28%	-69%	-99%
**Scn.6**	-8%	-61%	-99%
**Scn.7**	29%	-45%	-99%

**Fig 5:**
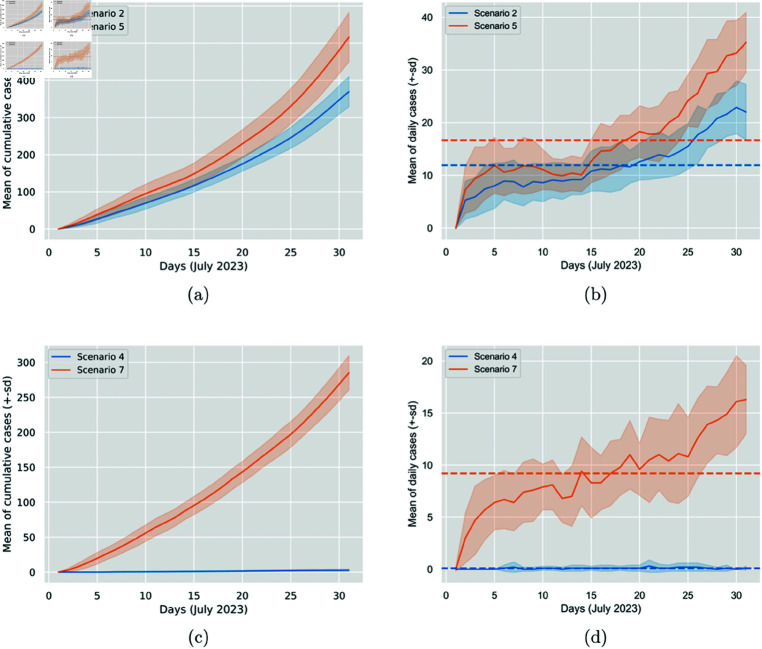
Comparison of the impact between N95 and surgical masks at 50% (Scenarios 2 and 5) and 100% (Scenarios 4 and 7) wearing percentages. The figure displays the cumulative generated cases per day and the daily generated cases. In Sub-figures (a) and (c), the solid line represents the 30-run mean of the cumulative generated cases per day, and the colourful area around it is the standard deviation. In Sub-figures (b) and (d), the solid line represents the 30-run mean of the daily generated cases, the colourful area around it is the standard deviation, and the dashed line is the average.

It is interesting to observe that while switching from 50% surgical mask to 50% N95 mask, the percentage reduction in average daily generated cases is 28%, when switching from 100% surgical mask to 100% N95 mask, the reduction shoots up to 99%. This observation reflects the remarkable effectiveness of N95, especially when worn by all participants (see Table 4 and Fig 5).

For the last mask-related experiment (*Experiment III*), we directly compared Scenarios 2-4 to study the effect of the wearing percentage while using the N95 mask, and Scenarios 5-7 for the surgical one. Again, all the t-tests returned very low p-values. Therefore, all wearing percentage changes significantly affect transmission for both mask types. As expected, a higher mask-wearing percentage results in a lower Covid spread.

The metrics and statistics reported for each involved scenario are shown in Table 3. It can be seen that for both mask types, a 100% wearing percentage reduces spread more than the respective 75%, which in turn reduces more than the 50% one. Table 5 summarises these conclusions while quantifying the effectiveness difference.

**Table 5:** Percentage changes in average daily generated cases when changing the mask-wearing percentage for the two mask types.

(a) N95 mask			(b) Surgical mask		
	Scn.3	Scn.4		Scn.6	Scn.7
Scn.2	-57%	-99%	Scn.5	-22%	-45%
Scn.3	-	-98%	Scn.6	-	-29%

Sub-table (a) displays percentage changes for the N95 mask, with changes from 50% (Scenario 2) to 75% (Scenario 3), from 50% (Scenario 2) to 100% (Scenario 4) and from 75% (Scenario 3) to 100% (Scenario 4). Sub-table (b) displays the respective changes for the surgical mask.

**Fig 6:**
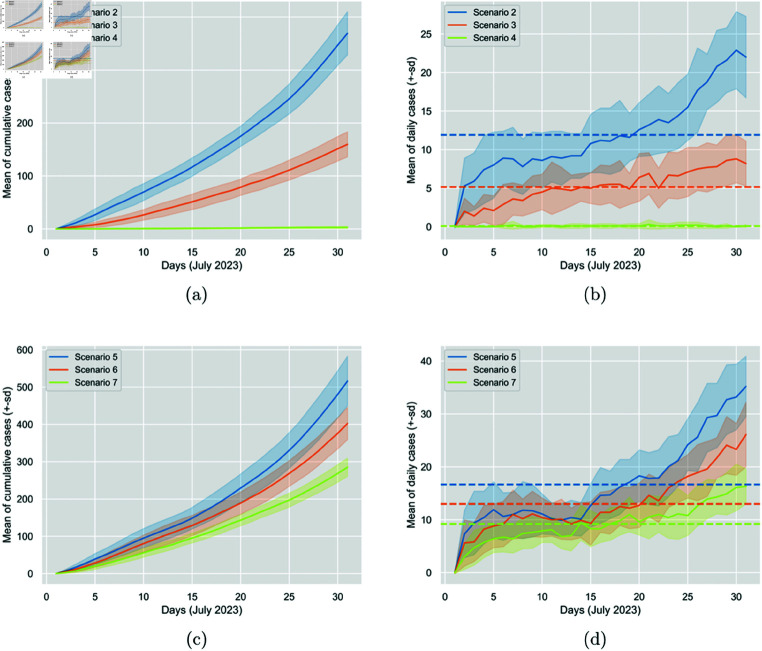
Comparison of the impact of increasing mask-wearing percentages from 50% (Scenarios 2 and 5) to 75% (Scenarios 3 and 6) to 100% (Scenarios 4 and 7) for N95 and surgical masks. The figure displays the cumulative generated cases per day and the daily generated cases. In Sub-figures (a) and (c), the solid line represents the 30-run mean of the cumulative generated cases per day, and the colourful area around it is the standard deviation. In Sub-figures (b) and (d), the solid line represents the 30-run mean of the daily generated cases, the colourful area around it is the standard deviation, and the dashed line is the average.

It is quite interesting to observe that for both mask types, increasing the mask-wearing percentage from 75% to 100% leads to a more significant reduction in cases than increasing from 50% to 75% (see Table 5). This is more prominent with the N95 mask. Referring back to Table 3, we can also observe that increasing the wearing percentage from 0% to 50% resulted in roughly the same reduction as the change from 50% to 75% did, despite the larger absolute difference in the percentages. This observation explains the significance of increasing mask-wearing percentages, while highlighting the importance of the higher ones, considering the multiplicative nature of such diseases and the infection chains caused by every infectious individual. Furthermore, mask type and efficacy play an essential role, as reflected by the more substantial reduction in cases observed when increasing the mask-wearing percentage for N95 masks compared to surgical ones. That is, changing from a lower to a higher wearing percentage seems to result in a more significant reduction when the N95 mask is used (see Table 5 and Fig 6). This effect is further amplified when considering the nearly 100% reduction in cases when all participants wear N95 masks.

### Vaccination

The effect of vaccination was studied considering the mask type factor for a fixed wearing percentage of 100%. Table 6 presents the metrics and statistics for 0%, 50%, and 100% vaccination percentages under three different mask-wearing conditions: N95 masks are worn by all individuals, surgical masks are worn by all individuals, and no mask is worn. All the t-tests performed for each comparison returned very low p-values, meaning that vaccination significantly affects and reduces transmission under all mask-wearing conditions and for all vaccination percentage changes.

**Table 6:** Metrics and statistics for Scenarios 8-10, 20-22, and 32-34.

	Scn.8	Scn.9	Scn.10	Scn.20	Scn.21	Scn.22	Scn.32	Scn.33	Scn.34
**Average daily generated cases**	0.2	0.1	0.0	14.8	9.3	1.5	37.9	24.9	5.1
**Cumulative generated cases**	6	3	0	489	303	48	1252	826	166
**Cumulative cases^a^**	122	118	115	589	405	160	1345	918	271
**Cumulative cases percentage**	1.4%	1.4%	1.4%	7.0%	4.8%	1.9%	16%	10.9%	3.2%
**Transmission ratio**	0.0	0.0	0.0	4.9	3.0	0.4	13.5	9.0	1.6

Vaccination percentage varies between 0%, 50% and 100% when N95, surgical, and no mask is used, respectively. ^a^ Includes both generated and exogenous cases (infectious entrants).

We can clearly observe that vaccination is an effective measure which becomes more critical when mask protection is weaker (either due to a mask with lower efficacy or no mask used). Increasing the vaccination percentage results in a more significant absolute reduction in such cases. More specifically, when the N95 mask is used, there is a statistically significant, but very limited reduction in cases between 0% and 100% vaccination percentage, given their already limited number. However, when the surgical mask is used, the cumulative generated cases are 489 (or 14.8 average daily) for 0% vaccination, 303 (9.3) for 50% and 48 (1.5) for 100%. When no mask is used, the respective numbers are 1252 (37.9), 826 (24.9) and 166 (5.1). It is, therefore, evident that the more vaccinated people, the fewer the generated cases, and especially when no other preventive measures are in place (in this case, masks), the vaccination impact is enormous.

**Fig 7:**
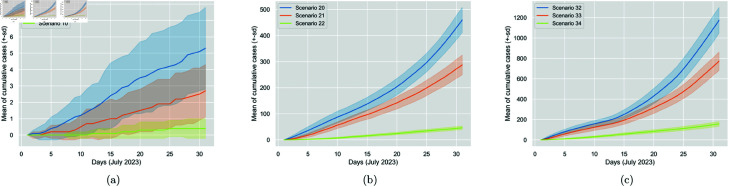
Comparison of the impact of increasing vaccination percentage from 0% (Scenarios 8, 20 and 32) to 50% (Scenarios 9, 21 and 33) to 100% (Scenarios 10, 22 and 34) for N95, surgical and no mask worn, respectively. The figure displays the cumulative generated cases per day. The solid line represents the 30-run mean, and the colourful area around it is the standard deviation.

It is also interesting to observe that, similarly to mask impact, increasing the vaccination percentage from 50% to 100% results in a more significant reduction in cases than increasing from 0% to 50%, as shown in Table 7. Changing from 50% to 100%, is almost equally as important as changing from 0% to 100%. This is the case for all mask-wearing conditions. The observation highlights the importance of higher vaccination percentages. Fig 7 displays the vaccination impact for the three mask-wearing conditions.

**Table 7:** Percentage changes in average daily generated cases when changing the vaccination percentage for the two mask types and no mask.

(a) N95 mask			(b) Surgical mask			(c) no mask		
	Scn.9	Scn.10		Scn.21	Scn.22		Scn.33	Scn.34
Scn.8	-50%	-100%	Scn.20	-37%	-90%	Scn.32	-34%	-87%
Scn.9	-	-100%	Scn.21	-	-84%	Scn.33	-	-80%

Sub-table (a) displays percentage changes for the N95 mask, with changes from 0% (Scenario 8) to 50% (Scenario 9), from 0% (Scenario 8) to 100% (Scenario 10) and from 50% (Scenario 9) to 100% (Scenario 10). Sub-table (b) displays the respective changes for the surgical mask, and Sub-table (c) for no mask.

### Ventilation

The effect of ventilation was studied considering the mask type factor for a fixed wearing percentage of 100%. Table 8 presents the metrics and statistics for 4 *ACH*, 8 *ACH*, and 12 *ACH* ventilation rates under the same three mask-wearing conditions. Based on the t-tests run, we conclude that ventilation does not significantly affect Covid transmission when N95 masks are worn by all individuals. However, it significantly affects transmission, when surgical or no mask is worn, for all ventilation rate changes.

**Table 8:** Metrics and statistics for Scenarios 11-13, 23-25, and 35-37.

	Scn.11	Scn.12	Scn.13	Scn.23	Scn.24	Scn.25	Scn.35	Scn.36	Scn.37
**Average daily generated cases**	0.1	0.1	0.1	11.7	9.4	8.3	33.0	25.4	22.1
**Cumulative generated cases**	2	3	2	383	307	269	1093	841	728
**Cumulative cases^a^**	116	116	120	485	408	373	1188	938	825
**Cumulative cases percentage**	1.4%	1.4%	1.4%	5.8%	4.8%	4.4%	14.2%	11.2%	9.8%
**Transmission ratio**	0.0	0.0	0.0	3.8	3.0	2.6	11.4	8.6	7.5

Ventilation rate varies between 4 *ACH*, 8 *ACH*, and 12 *ACH* when N95, surgical, and no mask is used, respectively. ^a^Includes both generated and exogenous cases (infectious entrants).

**Fig 8:**
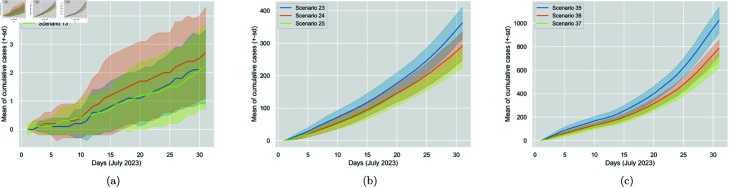
Comparison of the impact of increasing ventilation rate from 4 ACH (Scenarios 11, 23 and 35) to 8 ACH (Scenarios 12, 24 and 36) to 12 ACH (Scenarios 13, 25 and 37) for N95, surgical and no mask worn, respectively. The figure displays the cumulative generated cases per day. The solid line represents the 30-run mean, and the colourful area around it is the standard deviation.

It can be seen that ventilation is important, as higher rates reduce cases; however, its effect is not highly decisive. Table 9 shows the percentage changes in average daily generated cases. There is no impact when N95 masks are worn, as confirmed through t-tests results, and given the high effectiveness of N95 masks worn at 100%. Regarding the surgical mask and the no-mask condition, we observe similar percentage changes between the various rates, and as expected, increasing the ventilation rate from 4 *ACH* to 8 *ACH* results in a more significant reduction in cases (roughly 20%) compared to increasing the rate from 8 *ACH* to 12 *ACH* (roughly 10%). A more substantial reduction (roughly 30%) is observed when increasing the rate from 4 *ACH* to 12 *ACH*. Fig 8 displays the ventilation impact for the three mask-wearing conditions.

**Table 9:** Percentage changes in average daily generated cases when changing the ventilation rate for the two mask types and no mask.

(a) N95 mask			(b) Surgical mask			(c) no mask		
	Scn.12	Scn.13		Scn.24	Scn.25		Scn.36	Scn.37
Scn.11	0%	0%	Scn.23	-20%	-29%	Scn.35	-23%	-33%
Scn.12	-	0%	Scn.24	-	-12%	Scn.36	-	-13%

Sub-table (a) displays percentage changes for the N95 mask, with changes from 4 *ACH* (Scenario 11) to 8 *ACH* (Scenario 12), from 4 *ACH* (Scenario 11) to 12 *ACH* (Scenario 13) and from 8 *ACH* (Scenario 12) to 12 *ACH* (Scenario 13). Sub-table (b) displays the respective changes for the surgical mask, and Sub-table (c) for no mask.

### Distancing

**Fig 9:**
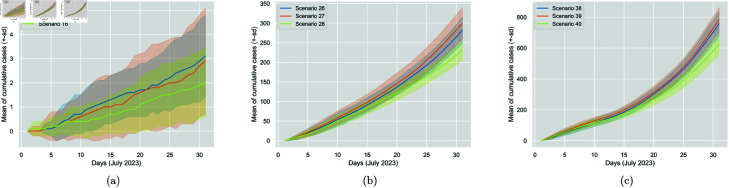
Comparison of the impact of increasing distancing rule rate from 0 m (Scenarios 14, 26 and 38) to 1 m (Scenarios 15, 27 and 39) to 2 m (Scenarios 16, 28 and 40) for N95, surgical and no mask worn, respectively. The figure displays the cumulative generated cases per day. The solid line represents the 30-run mean, and the colourful area around it is the standard deviation.

The effect of distancing was studied considering the mask type factor for a fixed wearing percentage of 100%. Table 10 presents the metrics and statistics for 0 *m*, 1 *m*, and 2 *m* distancing rules under the same three mask-wearing conditions. Based on the t-tests run, when all individuals wear N95 masks, there is no statistically significant impact on Covid transmission for a distancing rule increase from 0 *m* to 1 *m* or 1 *m* to 2 *m*. Transmission is only significantly affected when increasing the rule from 0 *m* to 2 *m*. However, this impact is not as important given the already low number of cases. Transmission is also not significantly affected when all individuals wear either the surgical mask or no mask, and a rule increase from 0 *m* to 1 *m* is attempted. However, when increasing the rule from 0 *m* to 2 *m* or 1 *m* to 2 *m*, a significant impact is noticed, i.e., a percentage change in cases of roughly -20% for both changes and mask-wearing situations. We, therefore, conclude that distancing is to some extent important, as a higher distancing rule reduces cases; however, its effect is not highly decisive, as reflected in Fig 9.

**Table 10:** Metrics and statistics for Scenarios 14-16, 26-28, and 38-40.

	Scn.14	Scn.15	Scn.16	Scn.26	Scn.27	Scn.28	Scn.38	Scn.39	Scn.40
**Average daily generated cases**	0.1	0.1	0.1	9.1	9.7	7.4	24.4	25.2	20.1
**Cumulative generated cases**	3	3	2	299	315	241	810	837	666
**Cumulative cases^a^**	115	120	114	404	414	342	903	933	760
**Cumulative cases percentage**	1.4%	1.4%	1.4%	4.8%	4.9%	4.1%	10.8%	11.1%	9%
**Transmission ratio**	0.0	0.0	0.0	2.9	3.2	2.4	8.7	8.7	7.0

The distancing rule varies between 0 *m*, 1 *m*, and 2 *m* when N95, surgical, and no mask is used, respectively. ^a^Includes both generated and exogenous cases (infectious entrants).

### Screening policy

The effect of the screening policy was studied considering the mask type factor for a fixed wearing percentage of 100%. Table 11 presents the metrics and statistics for 0%, 50%, and 100% screening percentages under the same three mask-wearing conditions. All the t-tests performed for each comparison returned very low p-values, indicating that the screening policy significantly affects transmission under all mask-wearing conditions and for all screening percentage changes.

**Table 11:** Metrics and statistics for Scenarios 17-19, 29-31, and 41-43.

	Scn.17	Scn.18	Scn.19	Scn.29	Scn.30	Scn.31	Scn.41	Scn.42	Scn.43
**Average daily generated cases**	0.2	0.1	0.0	11.0	9.5	2.5	29.1	24.8	9.0
**Cumulative generated cases**	5	3	0	359	313	83	957	820	307
**Cumulative exogenous cases**	210	114	11	185	99	10	181	93	11
**Cumulative cases^a^**	215	117	11	544	412	93	1138	913	318
**Cumulative cases percentage**	2.6%	1.4%	0.1%	6.5%	4.9%	1.1%	13.5%	10.9%	3.8%
**Transmission ratio**	0.0	0.0	0.0	1.9	3.2	8.1	5.3	8.8	28.2

Screening percentage varies between 0%, 50% and 100% when N95, surgical, and no mask is used, respectively.eak ^a^ Includes both generated and exogenous cases (infectious entrants).

**Fig 10:**
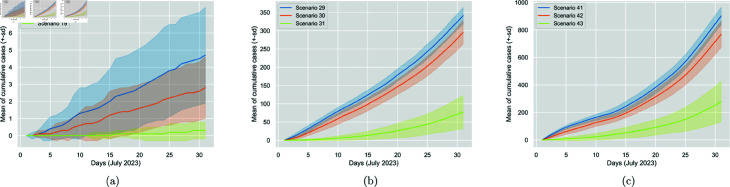
Comparison of the impact of increasing screening percentage from 0% (Scenarios 17, 29 and 41) to 50% (Scenarios 18, 30, 42) to 100% (Scenarios 19, 31 and 43) for N95, surgical and no mask worn, respectively. The figure displays the cumulative generated cases per day. The solid line represents the 30-run mean, and the colourful area around it is the standard deviation.

As anticipated, the higher the screening percentage, the fewer the exogenous cases (infectious entrants) and, therefore, the total of cases, regardless of the mask-wearing condition. The interesting metric here is the generated cases (average daily and cumulative), which decrease substantially with a high screening percentage, especially when all entrants are tested. More precisely, testing all the entrants is substantially more effective than testing none of them or even half of them, while similarly, testing half of them does not highly impact compared to not testing at all. Therefore, screening policy is highly decisive in mitigating the spread; however, a low testing percentage is not enough to significantly reduce cases. Table 12 summarises the percentage changes, and Fig 10 displays the screening policy impact while varying the screening percentages.

**Table 12:** Percentage changes in average daily generated cases when changing the screening percentage for the two mask types and no mask.

(a) N95 mask			(b) Surgical mask			(c) no mask		
	Scn.18	Scn.19		Scn.30	Scn.31		Scn.42	Scn.43
Scn.17	-50%	-100%	Scn.29	-14%	-77%	Scn.41	-15%	-69%
Scn.18	-	-100%	Scn.30	-	-74%	Scn.42	-	-64%

Sub-table (a) displays percentage changes for the N95 mask, with changes from 0% (Scenario 17) to 50% (Scenario 18), from 0% (Scenario 17) to 100% (Scenario 19) and from 50% (Scenario 18) to 100% (Scenario 19). Sub-table (b) displays the respective changes for the surgical mask, and Sub-table (c) for no mask.

## Discussion

In this paper we examined the effectiveness of some well-established Covid mitigation factors, and overall concluded a significant impact when they are in place. We found that mask-wearing might be the most important preventive measure given the significant reduction in cases reported, especially when the N95 mask is worn continuously by the entire population. Wearing surgical masks of lower efficacy significantly reduces transmission as well, but to a relatively limited extent. Therefore, we suggest establishing a mandatory N95 mask-wearing hospital policy, with the surgical mask as an alternative and no-mask situations only when unavoidable. When N95 masks are worn at 100%, our results suggest that vaccination is not critical. A high vaccination percentage substantially reduces transmission when the surgical mask is used, and especially when no mask is used. However, given that the mask-wearing assumptions discussed could not hold in many real-life situations, we believe all hospital entrants should be vaccinated even if N95 is used. For mask-wearing and vaccination measures, substantial mitigation is achieved only with high wearing and vaccination percentages, respectively. Screening policy is equally important, given the assumption of a 5% false negative ratio. We observed a massive decline in cases when the policy of 100% screening percentage was simulated; however, we acknowledge that this could be pretty impractical. A balanced and comprehensive screening policy is therefore recommended. Ventilation has been considered necessary, but not as crucial as mask-wearing, vaccination and screening policy. Nevertheless, a high ventilation rate could achieve a sound reduction in cases, reducing the respiratory airborne aerosol transmission probability, and is strongly suggested as a flawless preventive measure that could be combined with others without any trade-offs. Finally, we studied the distancing impact, assuming no violation of the distancing rule. No effect has been observed when the entire population wears the N95 mask; however, a limited effect has been concluded in other mask-wearing situations. One possible reason is that the rule does not apply to many hospital rooms, such as patient, emergency and surgery rooms, and does not affect the respiratory airborne aerosol transmission probability. However, it is suggested that people maintain a minimum distance of 2 *m* whenever possible, as indicated by the significant reduction in generated cases.

Our results are, overall, consistent with those of other similar agent-based modelling studies. While we might not be able to directly compare the effectiveness quantification (exact numbers), since the preventive measures are examined in different contexts in each model, we can still compare their broader impacts. For instance, Baccega *et al*. concluded a substantial percentage decrease in exposures in their modelled school when surgical masks are worn by all students and room ventilation measure is in place, being consistent with our respective results. They also demonstrated that by increasing the frequency and percentage of screening tests, Covid transmission is again significantly mitigated [1]. Macalinao *et al*. confirmed our findings regarding screening policy, suggesting that the ‘number of initially infected individuals’, i.e., in our case, the infectious entrants, substantially affect the eventual number of infections [3]. At the same time, considering various mask and vaccine types (and their efficacy), Ciunkiewicz *et al*. demonstrated a significantly longer elapsed time required for vaccinated mask-wearers to get infected, with the higher mask and vaccine efficacy resulting in less excess infection risk [7]. Finally, Hoertel *et al*. confirmed our results regarding mask-wearing and distancing, proving that combining the two NPIs could significantly reduce transmission [8].

This study has several limitations. First, despite the use of a relatively complex model that incorporates detailed mechanisms to approximate real-world dynamics, it still relies on simplifying assumptions that inevitably reduce the richness of reality, particularly in capturing the full complexity of human behaviour and interactions. These assumptions mainly include a constant ventilation rate in all hospital rooms, fixed positions and distances between individuals in some rooms, no variation in the number or identity of visitors among inpatients, no companions for outpatients, no violations of mask-wearing or distancing rules, no ‘hallway chats’, and no informal visits between staff members, patients, and visitors. As a result, the model may not fully reflect the nuanced and dynamic nature of real-case scenarios, especially under rapidly evolving epidemic conditions.

Second, although the model is parameterisable, the simulated hospital capacity is small and based on a simplified floor plan, which limits its immediate generalisability to larger health systems and may reduce the ability to draw accurate conclusions for real-world, large, and complex hospital environments.

Third, the probabilities governing the frequency and duration of various hospital-related events (such as admissions, discharges, doctor appointments, emergency medical visits, visits from visitors, movements and duration of staying in rooms), as well as the dynamic population at each time step, are based on assumptions rather than real-world data, which may affect the accuracy and realism of the simulation outcomes.

Future work could address these limitations by further relaxing assumptions related to human behaviour and interactions, and by incorporating larger-scale environments—potentially based on real hospital floor plan data—where events and dynamic population changes are linked to the floor plan and hospital capacity, based on empirical data and statistics.

At the same time, the model is somehow limited by the empirical findings of other models that drive our transmission mechanisms. However, adopting such models has enabled us to make our simulations more realistic and obtain more accurate results. Further enriching the transmission equations employed by incorporating factors we ignored, would be a worthwhile future extension. Such factors could be individuals’ sex, age, fitness level, and medical conditions or even increased inhalation rate due to heavier activities, increased virions concentration due to coughing or sneezing, room attributes like air sterilisation, and Virus characteristics as different variants, and so on. Similarly, a particular transmission driver of highly close contact, i.e., touching, hugging or kissing between individuals, could also be considered. These drivers could then be directly compared to identify the main one responsible for the infections.

Finally, the main factor under investigation was mask-wearing, while the remaining factors were studied in relation to it. This approach enabled a focused and thorough examination of the impact of mask type and mask-wearing percentage. However, the individual effects of the other factors were examined only under three conditions: with N95 or surgical mask worn at 100%, and with no mask. As a result, our study does not consider how multiple parameters may jointly affect transmission, nor does it capture all potential interdependencies between them. Future work could extend this analysis to explore combinatorial effects or even focus on other factors as well.

For instance, mask-wearing could be further studied by considering a mix of mask types being worn (as is usually the case), as well as related factors such as the duration and location of mask usage. In the same context, we could focus on infection patterns between different agent groups, such as between a non-mask-wearing visitor and a 50% mask-wearing inpatient. Additionally, this could be done by considering different values for the other factors (e.g., a 0% vaccination rate) instead of the fixed average used in our study. Screening policy could also be more thoroughly examined by considering policies of regularly testing admitted inpatients and staff members and quarantining them if needed. Observing the mitigation achieved under this more precise implementation would be interesting, as our simplification may have significantly influenced the perceived effectiveness of the measure.

## Conclusions

For this study, we modelled a typical hospital and examined how well-established factors, i.e., mask type and wearing percentage, vaccination percentage, ventilation rate, distancing rule, and screening policy, affect the transmission of Covid. In light of potential future outbreaks, we studied Covid within a critical environment so that more tailored and effective preventive measures can be immediately taken, driven by an enhanced understanding of the Virus spread in the hospital context. For this purpose, an ABM was developed to simulate the hospital setting’s dynamic nature and high complexity. While recognising that some assumptions could be relaxed, empirical data could be incorporated, and more combinations and factors could be explored in future extensions, we are confident that we have accurately quantified the influence of the main drivers of Covid transmission. Our results proved the importance and revealed the rationality behind the investigated factors’ implementation, while being tailored to the highly dynamic and distinct hospital environment. Therefore, hospital management and individuals should be aware of their potential and consider their feasibility for optimal adoption. Finally, an important part of the contribution lay in the methodological approach, which enables customisation to the hospital setting and offers a parameterisable tool that can be adapted to different diseases and healthcare environments.

## Supporting information

S1 File Appendix
(PDF)

S1 Fig Hospital floor plan as assumed in the model.
(TIFF)

S2 Fig Model visualization.
(TIFF)
