# Supplementary material for: Covid-19 in hospitals: Studying influencing factors through agent-based modelling
Source: PLoS One. 2025 Jun 18;20(6):e0326350. doi: 10.1371/journal.pone.0326350 (PMC12176126; doi:10.1371/journal.pone.0326350)
Supplement: S1 File — Appendix (PDF) [file pone.0326350.s001.pdf]

Appendix

Philippos Michaelides   Stefan Sarkadi

S1 Appendix. Source code structure and model usage guidelines

To use the model, execute the command `python main.py` in the source code directory. No command line arguments are required. Run the simulation through the appearing browser window.

Please note: In order for the code to run, you need to have Python 3.11.4 installed, along with the Mesa modular Python framework (version: 1.2.1). The Python libraries Matplotlib, Seaborn, Pandas, NumPy, Scipy, and tqdm are also required for full functionality.

The source code consists of 9 Python files and 2 CSV files:

1. `hospital.py`: Defines the general model's (or hospital's) and shared agents' methods. Most of the model parameters are also defined in this file regarding the hospital structure and policies, human behaviour and Covid. Please adjust them from the `init()` method. However, the relevant agent class defines the movement probabilities per agent type. Also, the fixed positioning rules per agent type in patient, emergency, office, surgery and 'out' rooms are defined in the `room.py` file. Some movement constraints are also enforced via the `getAvailableRooms()` method. The staff shifts are defined in the `defineStaffShifts()` method and the hospital traffic parameters (probabilities) in the `adjustTrafficParametersBasedOnTime()` method.

2. `staff.py`: Defines the parameters and methods related to the staff member agents (doctors, nurses, cleaners, receptionists). Adjust the movement probabilities per type from the `moveInNewRoom()` method and the movement constraints from the `getAvailableRooms()` method.

3. `patient.py`: Defines the parameters and methods related to the patient agents (inpatients and outpatients). Adjust the movement probabilities per type from the `moveInNewRoom()` method and the movement constraints from the `getAvailableRooms()` method.

4. `visitor.py`: Defines the parameters and methods related to the visitor agents. Adjust the movement probabilities per type from the `moveInNewRoom()` method and the movement constraints from the `getAvailableRooms()` method.

5. `room.py`: Defines the parameters and methods related to the room agents. Adjust the fixed positioning rules per agent type in patient, emergency, office, surgery and 'out' rooms from the `getPermittedPosition()` method.

6. `main.py`:

Section 1: Used to visualise the model on the browser. Adjust the rendered text details from the `render()` method and the colours used to visualise agents (rooms and humans) from the `agentPortrayal()` method. The investigated factors can be adjusted from the visualisation browser window.

Section 2: Used to run the model without visualising it, but rather with retrieving metrics, calculating and presenting hospital population and traffic statistics. These are stored in a file called *population_traffic_statistics.txt*.

Please comment out Section 1 while using Section 2 code, and vice versa.

`7. main2.py:` Used only for running the model (without visualisation), retrieving metrics, calculating and presenting Covid statistics. All our conclusions are drawn based on these results. Each scenario is run 30 times, and you can modify scenario details in the `scenarios` dictionary and adjust the number of runs by changing the relevant variable. Please specify the comparisons you want to make by adjusting the calls of the `compareScenarios()` method accordingly. To modify the visualisation details, adjust the `compareScenarios()` method. Please also note that only the investigated factors can be adjusted here. To modify other parameters, refer to the corresponding files discussed above. The results are stored in a file called *covid_statistics.txt*.

`8. verification.py:` Used to verify our Covid transmission equations as discussed in the 'Model verification' Section.

`9. graphs.py:` Used to manually visualise some results.

`10. building_data.csv:` Used to define the hospital structure (floor plan) and the maximum simultaneous occupancy for hospital rooms per agent type. A room's area is defined by its width and height, measured in cell grids towards the left and upwards, starting from a specific position (the bottom right corner), denoted as 'posX' and 'posY'. The hospital's width and height are defined in the `hospital.py` file.

`11. duration_limits.csv:` Used to define the duration ranges for each hospital room based on the agent type.

For all files, parameters and methods, detailed comments are included. Please refer to them for more information.

Please note that all probabilities, percentages, or efficacy levels should be given as decimal numbers between 0 and 1. For all the parameters, the values must be given in SI units (e.g., ventilation rates in ACH must be divided by 3600 to be converted to air changes per second).

S2 Appendix. Model description and assumptions

We model a typical medium-sized hospital, emphasising the key facilities, stakeholders, operations, and policies. The design decisions represent a simplified version of established practices that are followed in real-life cases, and they are fully parameterisable so that the model can adjust and capture various situations.

Environment

The layout and structure of the hospital building are simple. Apart from the doctor

offices and patient rooms with private toilets (double or single, totalling 32 beds), the building includes emergency rooms, surgery rooms, and toilet facilities. Additionally, the hospital features a spacious reception area, a cafe, a small staff kitchen, and the necessary corridors. We assume fixed positions for the individuals in offices, patient rooms, surgery rooms, and emergency rooms, meaning that patients, staff members and visitors are only allowed to hold a specific position within the room, depending on their role and room type. However, visitors in patient rooms and outpatients in offices, are placed by the defined distancing rule. Namely, the distancing rule defines a fixed distance between patients and visitors and between doctors and outpatients. For the rest of the rooms, all positions are available to be taken as long as this distancing rule is followed. The floor plan in Figure S1 illustrates all the hospital rooms and their dimensions.

Fig S1. Hospital floor plan as assumed in the model. The building measures 36 m in length, 42 m in width, has an assumed height of 3 m and consists of several rooms as labelled. Patient beds, emergency beds, surgery beds and examination chairs in patient, emergency, surgery and office rooms, respectively, indicate the inpatient or outpatient positions. Doctors, nurses and visitors are nearly positioned in fixed positions, according to the permitted rooms per role. Per the distancing rule, the entire space is available for the rest of the rooms.

Stakeholders and population

Regarding the individuals (stakeholders) who utilise the hospital, we can identify three distinct groups (roles): a) patients, who can be either inpatients or outpatients; b) staff, including doctors, nurses, cleaners, and receptionists; and c) visitors.

The population within the hospital is dynamic, with permanent changes observed in the patient and visitor population due to scheduled medical visits (doctor appointments), emergency medical visits, visits from visitors, admissions and discharges. Typically, at any given time between 07.00 and 19.00, there are approximately 25 inpatients (80% bed occupancy rate), 30 outpatients and 2 visitors. No visitors are inside the hospital at night, and the number of outpatients at any given time drops below five. These numbers remain relatively stable throughout the simulation. On the contrary, the staff population remains constant, with only temporary changes due to staff shifts. Staff population and shifts are summarised in Table S1.

We assume the absence of shift rotation, i.e., staff members have fixed assigned

Table S1. Staff population and shifts.

	07.00-13.00	13.00-15.00	15.00-19.00	19.00-22.00	22.00-07.00	Total population
**Doctor**	9	9	9	2	2	11
**Nurse**	30	20	20	10	10	60
**Cleaner**	4	2	2	2	1	7
**Receptionist**	4	2	2	2	1	7

working hours. This is to keep the model simple, given that Covid transmission is not affected by the individuals themselves but rather by their number. In the same context, no separation between weekdays and weekends is made; neither non-working (rest) days nor sick days are accounted for. Finally, no overtime is observed, and all staff members are assumed to leave the hospital when their shift ends, except for the medical staff involved in an ongoing surgery process, who conclude their duties after the surgery is completed.

To approximate hospital traffic, factors such as inpatient admissions and discharges, outpatient doctor appointments and emergency medical visits, as visits from external individuals, are determined based on probabilities triggered when people move between rooms, aiming to replicate the respective real-life rates. No differentiation based on weekday or year period is made. Table S2 displays the average daily traffic statistics achieved per period (day or night), which lead to a cumulative hospital population of around 8400 individuals over 31 days, including the constant staff members.

Table S2. Hospital traffic daily statistics. It shows how many patients and visitors, on average, enter the hospital per role and period.

	07:00-19:00	19:00-07:00
**Inpatient discharges**	8	0
**Inpatient admissions**	7	1
**Medical visits**[a]	250	4
**Visits from visitors**	10	0

[a] Doctor appointments and emergency medical visits.

Policies and human behaviour

All hospital policies are assumed to be strictly followed by the entire hospital population, influencing human behaviour.

Hospital operating hours are between 07:00 and 19:00 and include admissions, discharges, doctor appointments, emergency medical visits, surgical procedures, visiting hours, and cafe availability, with most hospital staff on duty. Outside these hours, only

emergency patient admissions and medical visits are permitted (at lower rates), while the hospital operates with a reduced staff. Namely, the cafe is closed, inpatient discharges and visits from visitors are not permitted (all visitors leave by 19.00), and surgical procedures are not conducted.

Each inpatient is assigned to a patient room until their discharge. Each double-patient room can accommodate a maximum of two inpatients, while each single-patient room can accommodate only one. We assume that the maximum bed occupancy of 32 is never exceeded. Inpatient visits between rooms are not allowed, and each inpatient can have only one constant visitor during their stay, dedicated to visits until discharge. At the same time, each visitor can only visit their respective patient, and visit frequency is assumed to be every one hour to one day. Also, outpatients are supposed to attend their doctor appointments or emergency medical visits alone. Furthermore, each doctor has an office assigned exclusively to them, which is shared only with doctors of different shifts. Visits between doctors in their offices are not permitted. Regarding the surgery process, we assume that exactly two doctors and four nurses are strictly required. We also expect all staff members, along with the patient, to enter and leave the surgery room together. Finally, cleaners are restricted from entering the surgery room, emergency rooms and offices while medical treatment is delivered. Table S3 summarises the allowed rooms and facilities per role, including the maximum simultaneous occupancy for each room, which restricts the movement of individuals between rooms along with the distancing rule where applicable.

To realistically model human behaviour inside the hospital, additional behavioural rules are enforced. Individuals generally move between their permitted rooms based on specific probabilities, incorporating predefined stay duration ranges within them to emulate lifelike behaviour. Emphasis is given to behavioural details, such as doctors and nurses entering patient rooms, emergency rooms, and surgery rooms only when patients are inside. They also specifically only visit occupied beds when attending these rooms. Similarly, outpatients visit doctor offices, and visitors enter patient rooms only when the doctor and the inpatient, respectively, are inside. At the same time, visitors always visit their respective patients when they come to the hospital and permanently stop visiting the hospital upon their discharge. Assuming patient rooms include private toilets, inpatients do not use other toilet facilities and do not leave their rooms during

Table S3. Permitted hospital rooms and maximum simultaneous occupancy per role.

	Inpat.	Outpat.	Doctor	Nurse	Cleaner	Recept.	Visitor
**Patient room**	1 or 2	-[a]	1 or 2	1 or 2	1	-	1 or 2
**Office**	-	1	1	-	1	-	-
**Emergency room**	2	2	4	4	1	-	-
**Surgery room**	1	-	2	4	1	-	-
**Cafe**	Un.[b]	Un.	Un.	Un.	Un.	Un.	Un.
**Toilet**	-	Un.	Un.	Un.	1	Un.	Un.
**Kitchen**	-	-	Un.	Un.	1	Un.	-
**Reception**	Un.	Un.	Un.	Un.	Un.	Un.	Un.
**Corridor**	Un.	Un.	Un.	Un.	Un.	Un.	Un.

[a] Restricted room.
[b] Unlimited occupancy.

the night. Finally, corridor rooms are utilised based on a given probability (assuming not for 'hallway chats'), and depending on the room type, individuals may move within their rooms rather than remaining in a static position to achieve a more authentic movement behaviour.

The duration ranges and movement choices (probabilities) depend on the individual role and room type, as illustrated in Table S4 and Table S5, respectively. Important to note that the probabilities are triggered only when the individual has spent the randomly chosen duration inside the room and is ready to move to a new one. Specifically, upon the end of the period, the decision to move to a new room is considered based on these movement probabilities. No movement is performed if the same room is selected again. Also, the movement decision may not be applied if it violates the maximum simultaneous occupancy of the selected (next) room, the distancing rule, or the hospital policies and behavioural rules described. This might, in practice, lead to different movement probabilities at different simulation stages. For instance, according to the hospital policy of the cafe opening hours, it is not allowed to visit the cafe at night. Therefore, even if selected, there is zero probability of going to the cafe during nighttime. The same holds for other policies (e.g., no surgery operations during the night), or when entering a room would violate the distancing rule inside or exceed its maximum simultaneous occupancy.

At the same time, we presume that discharged inpatients and outpatients that have

Table S4. Duration ranges (in minutes) per room and role.

	Inpat.	Outpat.	Doctor	Nurse	Cleaner	Recept.	Visitor
**Patient room**	10-100	-[a]	1-5	1-10	5-10	-	10-40
**Office**	-	10-40	10-100	-	5-10	-	-
**Emergency room**	5-20	5-20	1-5	5-20	5-10	-	-
**Surgery room**	30-120	-	30-120	30-120	5-10	-	-
**Cafe**	1-15	1-15	1-15	1-15	5-10	1-15	1-30
**Toilet**	-	1-5	1-5	1-5	5-10	1-5	1-5
**Kitchen**	-	-	1-5	1-5	5-10	1-5	-
**Reception**	1-40	1-40	1-5	1-5	5-10	10-100	1-5
**Corridor**	1-3	1-3	1-3	1-3	5-10	1-3	1-3

[a] Restricted room.

Table S5. Movement probabilities per room and role.

	Inpat.	Outpat.	Doctor	Nurse	Cleaner	Recept.	Visitor
**Patient room**	0.75	0.0	0.2	0.1	0.4	0.0	0.55
**Office**	0.0	0.4	0.45	0.0	0.1	0.0	0.0
**Emergency room**	0.05	0.2	0.05	0.3	0.05	0.0	0.0
**Surgery room**	0.05	0.0	0.0[a]	0.0[a]	0.05	0.0	0.0
**Cafe**	0.05	0.05	0.05	0.05	0.05	0.05	0.1
**Toilet**	0.0	0.05	0.05	0.05	0.1	0.05	0.05
**Kitchen**	0.0	0.0	0.05	0.05	0.05	0.05	0.0
**Reception**	0.0	0.05	0.05	0.3	0.1	0.75	0.05
**Corridor**	0.1	0.1	0.1	0.15	0.1	0.1	0.1
**Leaving**	0.0[b]	0.15	0.0[c]	0.0[c]	0.0[c]	0.0[c]	0.15

[a] Visiting the surgery solely depends on the patient's respective movement decision.
[b] Leaves the hospital on discharge.
[c] Leaves the hospital on shift change.

concluded their medical visit and departed from the hospital, do not return within the simulation. Patients and visitors entering the hospital start from either the reception area or the cafe, while staff members, after shift change, could be located in any permitted room (excluding the surgery room), depending on their role (except doctors that always start from their office). The probabilities of initial placement in the hospital per role, for either new patients and visitors or returning visitors and staff members, are summarised in Table S6.

In general, a realistic hospital routine is achieved in terms of frequency, sequence and duration of activities, while still experiencing some unrealistic or unusual cases, such as

Table S6. Initial placement probabilities per room and role.

	Patient	Doctor	Nurse	Cleaner	Recept.	Visitor
**Patient room**	0.0	0.0	0.5	0.45	0.0	0.0
**Office**	0.0	1.0	0.0	0.1	0.0	0.0
**Emergency room**	0.0	0.0	0.2	0.05	0.0	0.0
**Surgery room**	0.0	0.0	0.0	0.0	0.0	0.0
**Cafe**	0.3	0.0	0.05	0.05	0.05	0.3
**Toilet**	0.0	0.0	0.05	0.1	0.05	0.0
**Kitchen**	0.0	0.0	0.05	0.05	0.05	0.0
**Reception**	0.7	0.0	0.1	0.1	0.75	0.7
**Corridor**	0.0	0.0	0.05	0.1	0.1	0.0

outpatients visiting both doctor offices and emergency rooms. However, these cases do not affect Covid transmission and, therefore, do not impact the results and conclusions drawn. Attempting to further or completely avoid such cases would significantly increase the model's complexity, decrease its readability and maintainability, and increase the required execution time.

External environment

While the model primarily focuses on the dynamics within the hospital setting, the external environment is not entirely ignored. We expect that an individual entering the hospital could be infected, based on a given probability, assuming that in all these cases, they are in the Covid stage able to transmit the Virus (they are infectious), to account for the worst-case scenario. We also assume that this probability is the same for every entrant, irrespective of potential influencing factors. For this study, we assume a fixed probability of 2%, while acknowledging that, generally, it should be determined based on the specific Covid situation at the time of assessment.

S3 Appendix. Covid transmission

Covid is a respiratory disease primarily spread through respiratory infectious particles of various sizes exhaled from infectious individuals while breathing, speaking, coughing and sneezing, leading to airborne transmission [1]. Infectious particles contain smaller virus particles (virions) [2], currently defined based on size. The larger particles (at least 5 or 10 μm in diameter) are termed droplets, and the smaller aerosols [3]. Generally, they range from 5 to 500 μm for droplets and smaller than 5 μm for

aerosols [1].

Released infectious particles travel through the air until they either fall into the ground or on a surface, or get inhaled. Those landed on surfaces could infect other individuals touching them. However, the risk of infection from touching a contaminated surface is generally considered low (less than 5 or even 1 in 10,000 contacts) [4, 5]. At the same time, people can and do avoid touching surfaces to protect themselves, and therefore, we do not consider surfaces as a driver of exposure in our analysis. Likewise, assuming that people are warned and careful, we do not consider the probability of contracting the Virus through touching, hugging or kissing.

On the contrary, it has been proven that inhaled infectious particles released into the air can easily infect those who inhale them. This type of airborne transmission is influenced by the size of the particles [1]. That is, larger particles (i.e., large droplets) settle quickly due to gravity and are more likely to cause infection between individuals in close proximity, while smaller particles (i.e., aerosols) can remain suspended in the air for several minutes or hours, making it possible for people to become infected even when they are not near an infectious person [4].

S4 Appendix. Model implementation

For the model implementation, the Python programming language is used (version: 3.11.4), along with the modular Python framework of Mesa (version: 1.2.1). Python's high-level nature, simplified syntax and extensive support libraries that facilitate programming complicated tasks, made the programming language decision straightforward. Consequently, the Mesa framework was selected as a user-friendly way to develop ABMs, providing modelling, analysis and visualisation components.

The hospital building and rooms are represented on a 2D grid, where each grid cell is assumed to be 1 m in width and height. This provides the total width and height of each room and the entire hospital, as well as the measures of area and volume used for calculating Covid transmission probabilities (assuming a third dimension of 3 m). Mesa's 'Grid' ('MultiGrid') significantly facilitates agent movements and consequently helps simulate behaviours and enforced hospital policies, by retrieving important metrics, such as the distance between agents or the population of a given grid area. These metrics are also helpful in triggering the Covid transmission equations.

Both hospital rooms and humans are considered agents and are represented as data structures, instances of classes (objects) with specific data (attributes) and behaviours (methods) depending on their role (class/type), that simulate their values and behaviour, respectively. This design choice enables polymorphism, i.e., an efficient and effective way to simulate and achieve the desired behaviour of each agent type by using the same method names (signatures) and different implementations. This is a key given that each agent type behaves differently and adheres to different constraints.

A 'Hospital' class and a 'hospital' instance are responsible for defining the hospital area (grid) and initialising all the agents (rooms and humans), implementing in this way both the initial agent placement and the new arrivals (inpatient admissions, outpatient medical visits and visitor visits). The 'hospital' instance also triggers all the agents' next actions (calling their 'step' method) while holding and updating the simulation clock or other global hospital metrics like the current positive cases or hospital population. Methods that drive the hospital's overall behaviour and policies (e.g., conducting a surgical procedure) are also implemented.

A 'step' method is defined for each agent type, responsible for determining the next action, movement or behaviour in general, updating values and triggering probabilities based on the type, simulation progress and dynamics. For this, adding each agent to the global Mesa 'scheduler' is required, determining the order in which they act and the space for them to move around.

Therefore, the movement of agents inside the hospital corresponds to movement on the grid, while an ample grid space (implemented as a particular room) is allocated to simulate the external environment, where staff off duty and visitors not currently on a visit are located. Moving an agent to a random permitted room is randomly determined based on the movement probabilities, while moving an agent to a specific room is implemented by adjusting these probabilities accordingly. Each room has its defined area to differentiate agents into different rooms, which is useful for managing and maintaining room data such as the current population of the room, the current infectious agents population, the currently available positions, and so on.

Finally, a 'main' class is responsible for initialising the 'hospital' instance, performing the statistical analysis, and presenting and storing the results. For this purpose, the Matplotlib, Seaborn, Pandas, NumPy and SciPy Python libraries are used.

The 'main' class is also used to visualise the model in the browser, using the Mesa visualisation package, as shown in Figure S2.

Fig S2. Model visualisation. Each grid cell is assumed to be 1 m in width and height. Based on the object types, rooms are visualised as rectangles (as their labels indicate) and humans as dots of different colours. We use cyan for inpatients, pink for outpatients, dark blue for doctors, grey for nurses, green for cleaners, yellow for receptionists and orange for visitors. Regardless of their role, red is used for *infected* individuals and white for the *exposed* ones, as defined by the SEIR model.

Important to mention that great emphasis is given to implementation efficiency to achieve the lowest possible computational complexity. For instance, when updating the population of infectious agents in each room, instead of looping through all possible positions and assessing the Covid status of each occupant, a population variable is maintained and updated accordingly. This is crucial considering the already high complexity required due to the complicated behaviour we want to achieve, and especially because of the large number of simulation runs we need to perform.

S5 Appendix. Simulation

Hardware

All simulations run on a Google Compute Engine Virtual Machine (n2-standard-8 instance) with the following technical specifications: OS: Ubuntu 20.04.6 LTS x86_64, Kernel: 5.15.0-1038-gcp, CPU: Intel Xeon (8) @ 2.800GHz, Memory: 316MiB / 32089MiB (around 32GB).

Initial Model State

The initial state of the model, before any actions or movements are executed, can be described as follows: we define 25 inpatients, 30 outpatients, and 10 visitors to be initially located inside the hospital. Also, all staff members of the first shift are assumed to perform their duties normally. The starting agent positioning follows all the hospital policies and human behaviour adopted, adhering to the permitted rooms per role, maximum simultaneous occupancy of rooms, and the distancing rule applied. Additionally, doctors start from their offices, visitors do not start from patient rooms, and inpatients do not start from the surgery room. The probability of each agent starting as infectious is 2%.

S6 Appendix. Experimental set-up

a. Mask type and wearing percentage: We only account for the N95 and surgical mask types, with efficacy levels of 99% and 59%, respectively. We also consider wearing percentages of 0%, 50%, 75% and 100%.

Experiment I: To study the mask impact, we use a baseline scenario in which no mask is worn, and average values are assumed for all other factors (Scenario 1). Scenario 1 is used for an independent comparison with Scenarios 2-7, where the mask is used on different conditions (in terms of mask type and wearing percentage) to quantify mask impact under each of these conditions. We set up a first hypothesis test as follows:

Null hypothesis (H_0): There is no significant difference in Covid transmission between wearing and not wearing a mask.

Alternative hypothesis (H_1): There is a significant difference in Covid transmission between wearing and not wearing a mask.

By independently comparing Scenarios 2-7 with Scenario 1, we can determine the conditions under which H_0 is rejected in favour of the alternative. That is, we can identify the specific mask-wearing conditions in which masks effectively reduce Covid transmission.

Experiment II: In addition, the two mask types are directly compared to each other under all three conditions of mask-wearing percentage. This is important to comprehend whether the higher efficacy of the N95 mask significantly reduces Covid transmission, compared to the surgical mask, and to quantify this difference in effect in relation to the essential factor of wearing percentage. For this study, a second hypothesis test is set up as follows:

H_0: There is no significant difference in Covid transmission between the N95 and surgical masks.

H_1: There is a significant difference in Covid transmission between the N95 and surgical masks.

To determine the conditions (mask-wearing percentages) under which H_0 is rejected in favour of the alternative, all Scenarios 2-4 are independently compared with all Scenarios 5-7. Consequently, we can identify the wearing percentages in which the N95 mask effectively reduces Covid transmission more significantly than the surgical mask.

Experiment III: Finally, to directly compare the wearing percentage impact when the two mask types are worn, two more hypothesis tests are set up as follows:

H_0: There is no significant difference in Covid transmission between the different mask-wearing percentages when the N95 mask (or the surgical mask, respectively) is worn.

H_1: There is a significant difference in Covid transmission between the different mask-wearing percentages when the N95 mask (or the surgical mask, respectively) is worn.

To determine under which conditions (mask-wearing percentages or, more precisely, wearing percentage changes) H_0 is rejected in favour of the alternative, Scenarios 2-4 (and Scenarios 5-7, respectively) are independently compared with each other, so we can observe the effect of a higher percentage while using a specific mask type.

b. Remaining factors: To study the effect of the remaining factors (vaccination, ventilation, distancing, and screening policy), four last hypothesis tests are set up as follows:

H_0: Factor f, where f is a remaining investigated factor, does not significantly affect Covid transmission.

H_1: Factor f, where f is a remaining investigated factor, does significantly affect Covid transmission.

For each f, to determine under which factor-related conditions (i.e., vaccination percentage changes, ventilation rate changes, distancing rule changes or screening percentage changes, depending on f) H_0 is rejected in favour of the alternative, the respective scenarios are compared to each other (*Experiment IV*). Given that we are interested in studying the effects considering mask type, we conduct each hypothesis test for both N95 and surgical masks (maintaining a fixed wearing percentage of 100% to study the impact in an absolute case) and for no mask worn as well. Therefore, for each f, H_0 might be rejected for none, some, or all mask-wearing conditions.

For all the hypothesis tests, we aim to determine whether there is a statistically significant difference between the statistics (means) resulting from the different scenarios, allowing us to reject H_0. Multiple independent samples t-tests (two-sample t-tests) are performed (one for each comparison), given that all t-test assumptions and requirements are met [6]. Specifically, our ratio scale data groups are obtained through independent and unrelated runs with appropriately implemented and consistent randomness. They are also normally distributed based on the Central Limit Theorem,

given that we simulate 30 runs for each scenario [7]. We also check the groups' variances performing the Levene's test [8] before conducting the t-test, so that the unequal variances t-test (or Welch's t-test) [9] is chosen in case of not homogeneous variances, to account for this requirement as well. For our t-tests, a significance level (alpha) of 0.05 is used. That is, the probability of erroneously rejecting H_0 (false positive) is 5%, which is considered sufficient for the context and purpose of our study.

We consider rejecting the H_0 only based on the average daily generated cases. That is to say, we only perform t-tests based on the average daily generated cases of the two compared scenarios, aiming to determine whether the means of the averages from 30 runs of each scenario are significantly different.

References

1. Anand S, Mayya Y. Size distribution of virus laden droplets from expiratory ejecta of infected subjects. Scientific reports. 2020;10(1):1–9.

2. Burrell CJ, Howard CR, Murphy FA. Virion structure and composition. Fenner and White's Medical Virology. 2017; p. 27.

3. Randall K, Ewing ET, Marr LC, Jimenez J, Bourouiba L. How did we get here: what are droplets and aerosols and how far do they go? A historical perspective on the transmission of respiratory infectious diseases. Interface Focus. 2021;11(6):20210049.

4. Science Brief: SARS-CoV-2 and Surface (Fomite) Transmission for Indoor Community Environments; 2021. Centers for Disease Control and Prevention `https://stacks.cdc.gov/view/cdc/104762`.

5. Lewis D, et al. COVID-19 rarely spreads through surfaces. So why are we still deep cleaning. Nature. 2021;590(7844):26–28.

6. Kim TK, Park JH. More about the basic assumptions of t-test: normality and sample size. Korean journal of anesthesiology. 2019;72(4):331–335.

7. Lind DA, Marchal WG, Wathen SA. In: Statistical techniques in business and economics. 15th ed. McGraw-Hill/Irwin; 2012. p. 279–280.

8. Gastwirth JL, Gel YR, Miao W. The Impact of Levene's Test of Equality of Variances on Statistical Theory and Practice. Statistical Science. 2009;24(3):343 – 360. doi:10.1214/09-STS301.

9. Zimmerman DW, Zumbo BD. Rank transformations and the power of the Student t test and Welch t'test for non-normal populations with unequal variances. Canadian Journal of Experimental Psychology/Revue canadienne de psychologie expérimentale. 1993;47(3):523.
