# Supplementary figures and images for: Covid-19 in hospitals: Studying influencing factors through agent-based modelling
Source: PLoS One. 2025 Jun 18;20(6):e0326350. doi: 10.1371/journal.pone.0326350 (PMC12176126; doi:10.1371/journal.pone.0326350)

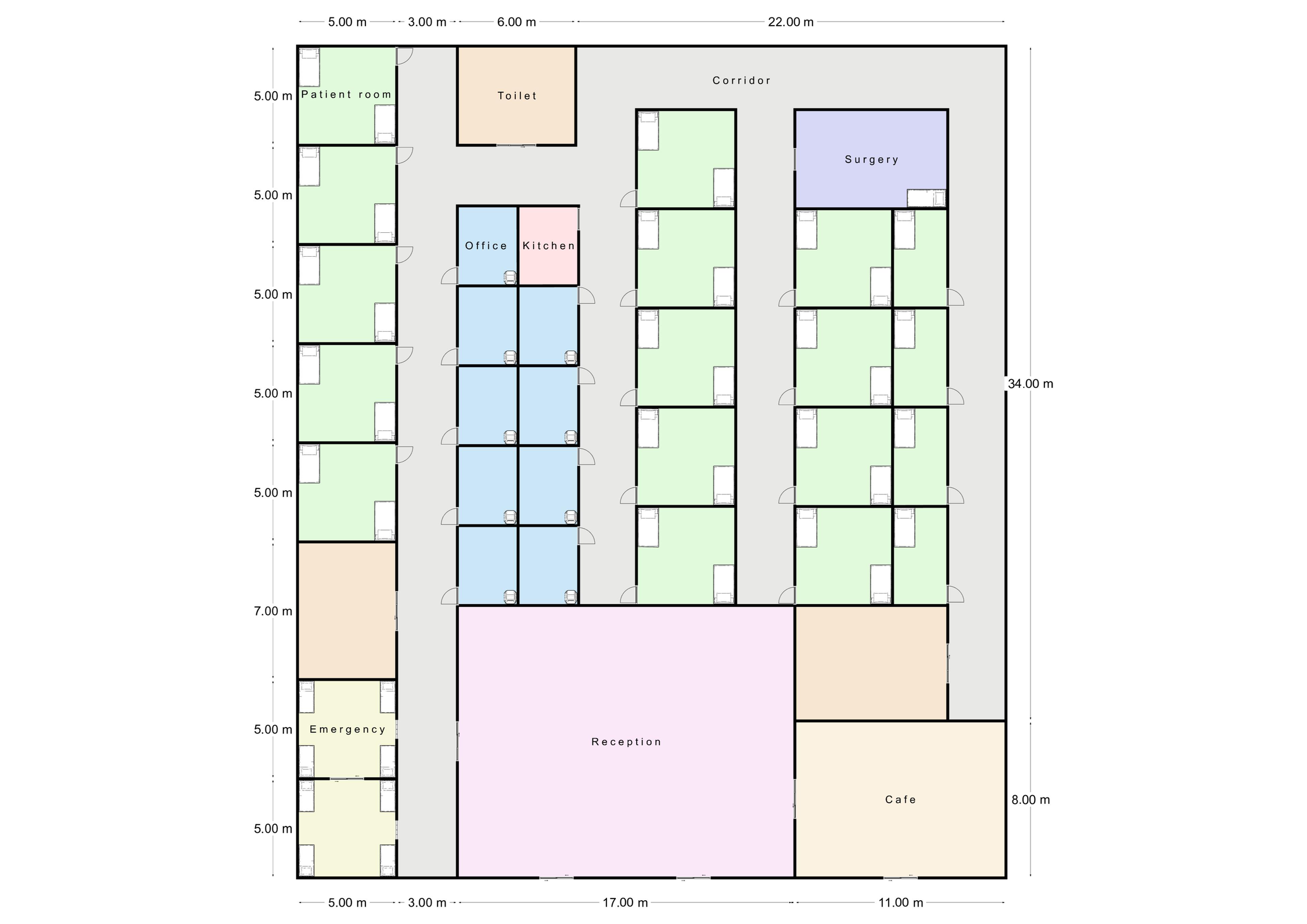

Supplement: S1 Fig — Hospital floor plan as assumed in the model. (TIFF) [file pone.0326350.s002.tif]

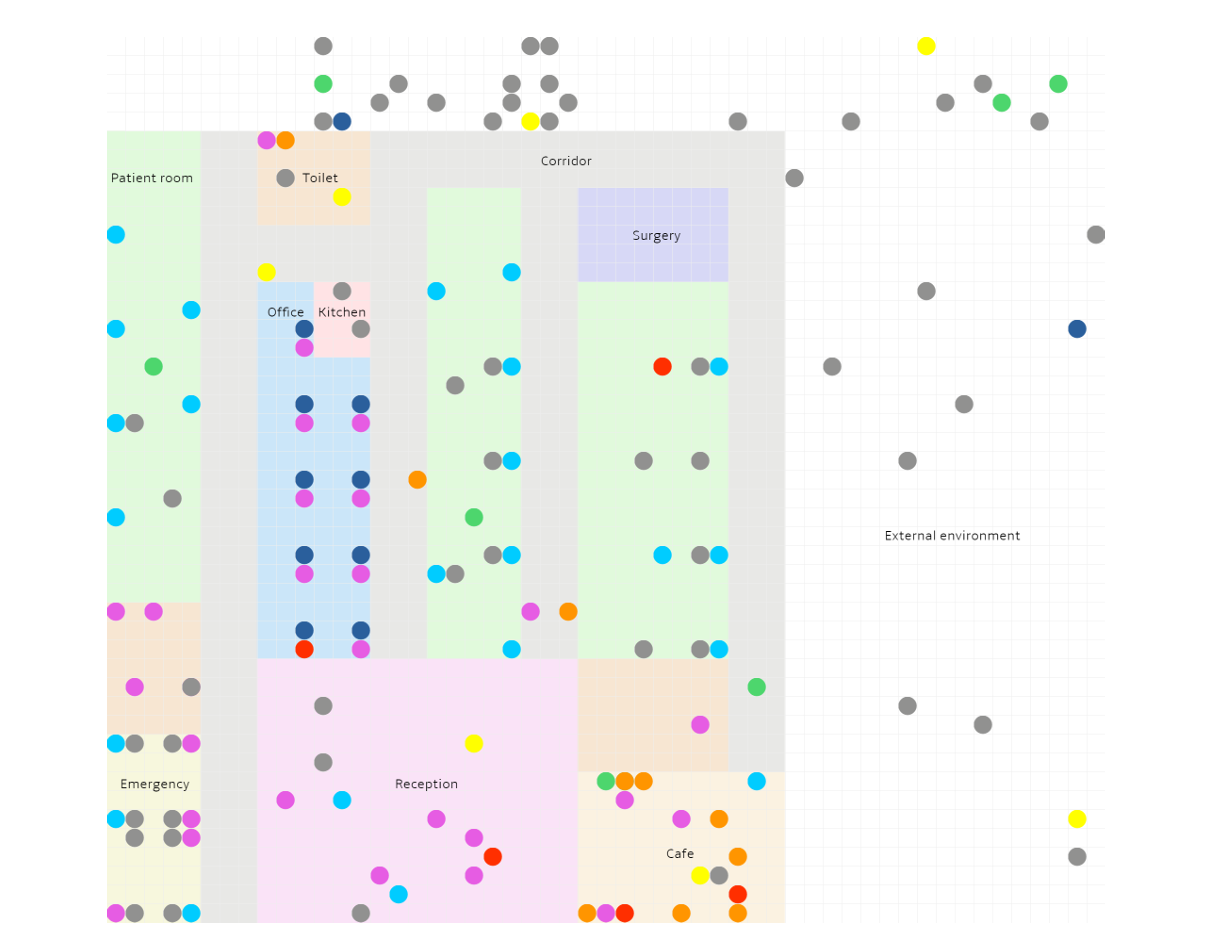

Supplement: S2 Fig — Model visualization. (TIFF) [file pone.0326350.s003.tif]
